# A systematic review and meta-analysis of studies exploring prevalence of non-specific anxiety in undergraduate university students

**DOI:** 10.1186/s12888-023-04645-8

**Published:** 2023-04-11

**Authors:** Irtiqa Ahmed, Cassie M. Hazell, Bethany Edwards, Cris Glazebrook, E. Bethan Davies

**Affiliations:** 1 School of Medicine, University of Nottingham, Nottingham, UK; 2 Department of Psychological Interventions, University of Surrey, Guildford, UK; 3 School of Social Sciences and Humanities, University of Westminster, London, UK; 4 Clinical Neurosciences and Mental Health, School of Medicine, Institute of Mental Health, University of Nottingham, Nottingham, UK; 5 NIHR MindTech MedTech Co-Operative, Institute of Mental Health, School of Medicine, The University of Nottingham, Nottingham, UK

**Keywords:** Anxiety, Meta-analysis, Prevalence, Students, Systematic review

## Abstract

**Background:**

Anxiety is a common mental health problem in the general population, and is associated with functional impairment and negative impacts upon quality of life. There has been increased concern about university students’ mental health in recent years, with a wide range of non-specific anxiety rates reported worldwide in undergraduate university students. We aimed to explore prevalence of non-specific anxiety in undergraduate university student populations.

**Methods:**

Four databases were searched to identify studies published between 1980 and 2020 which investigated prevalence of non-specific anxiety in undergraduate university students. Each study’s quality was appraised using a checklist. Sub-analyses were undertaken reflecting outcome measure utilized, course of study, location of study, and whether study was before or during the COVID-19 pandemic.

**Results:**

A total of 89 studies – representing approx. 130,090 students—met inclusion criteria. Eighty-three were included in meta-analysis, calculating a weighted mean prevalence of 39.65% (95% CI: 35.72%—43.58%) for non-specific anxiety. Prevalence from diagnostic interview studies ranged from 0.3%-20.8% 12-month prevalence. Prevalence varied by outcome measure used to assess non-specific anxiety, the type of course studied by sample, and by study location. In half the studies, being female was associated with being more likely to have higher non-specific anxiety scores and/or screening above thresholds. Few of the included studies met all quality appraisal criteria.

**Conclusion:**

The results suggest that approximately a third of undergraduate students are experiencing elevated levels of non-specific anxiety. Results from sub-analyses have identified some methodological issues that need consideration in appraising prevalence in this population.

**Supplementary Information:**

The online version contains supplementary material available at 10.1186/s12888-023-04645-8.

## Introduction

Anxiety disorders are mental health disorders characterised by the presence of anxiety, hyper-arousal, and fear; and are often accompanied by other physical and cognitive symptoms, such as insomnia, restlessness, and concentration difficulties [1]. These symptoms cause significant distress, functional impairments and reduced quality of life [2, 3]. Anxiety disorders are relatively common with approximately 284 million people experiencing anxiety at any one time worldwide [3]. Anxiety disorders are more prevalent in females than males across the lifespan, with generalised anxiety disorder having an estimated lifetime prevalence of 3.7% [3–5].

The pervasiveness of anxiety disorders has prompted investigation of its prevalence amongst specific sub-populations. With recent media reports reporting a ‘student mental health crisis’ [6], the mental health of university students has received greater attention in recent years. A review by Sheldon and colleagues [7] found both depression and suicide-related outcomes are pervasive amongst university students with a pooled prevalence of 21%. However, anxiety though is one of the most commonly reported mental health problems experienced by university students [8], yet equivalent pooled estimates are not available. Findings from the most recent Healthy Minds study [9] found almost a third of university students in the United States screened for a possible anxiety disorder. Higher levels of anxiety in students have been associated with lower academic performance [10, 11]. Although the majority of the media attention on this subject has been in Western countries [6], this issue is not isolated to English-speaking universities. Evidence shows that poor mental health is also common amongst students studying in Asia, Africa, and the Middle East for example [3–15].

The high rate of anxiety disorders amongst students is considered to be in part a product of the high-risk late adolescence-early adulthood developmental phase that most students are at when they begin their studies [16] but is more likely an artefact of the current socio-economic context. For example, young people are facing more judgment and higher expectations from society within increasingly competitive environments compared to that of previous generations [17]. Additionally, an unintended consequence of active outreach and widening participation efforts to make Higher Education more accessible is the increased number of students attending university who are at increased risk for poorer mental health (e.g., those from lower socioeconomic status, ethnic minorities, or those with additional support needs) [16].

Given the increasing concern about the mental health of university students, it is important to consider what the current evidence is around the prevalence of specific mental health disorders - like anxiety - in this population. There are several systematic reviews exploring prevalence of non-specific anxiety in medical and nursing student populations [14, 18–20], collectively reporting a prevalence of 32% or higher. We cannot assume that these findings can be generalised to the wider student population, however, due to the distinct features of healthcare courses; including the time [21] and emotional intensity [22] of their studies, and concerns around being seen as ‘fit to practice’ [23]. To our knowledge there has been no synthesis of studies examining the prevalence of non-specific anxiety in the wider undergraduate university student population. The present review will address this gap in the literature.

We aimed to identify and meta-analyse studies reporting a prevalence for non-specific anxiety symptoms among undergraduate university students. The secondary aims of this review were to synthesise reported socio-demographic differences in prevalence of non-specific anxiety, and to explore trends in non-specific anxiety prevalence over time.

## Methodology

### Search strategy and study eligibility

A systematic review was performed to identify English language peer-reviewed studies published between 1^st^ January 1980 and 30^th^ September 2020 (PROSPERO registration: 2020 CRD42020213088). We used a search string reflecting non-specific anxiety, prevalence, and university students, which were developed through reviewing previous relevant systematic reviews into university students’ mental health: [College students OR university students OR undergraduate students OR medical students OR undergraduate medical students OR undergrad*] AND [Anxiety OR generalized anxiety OR general anxiety OR generalized anxiety disorder OR anxiety disorders] AND [Prevalence OR incidence]. When devising our search terms, we reviewed previous reviews on the target population or the target outcome. We included medical students in our review as we were interested in the prevalence of anxiety across all Higher Education students. The inclusion of ‘anxiety’ and ‘generalised anxiety’ terms were selected to reflect our focus on non-specific anxiety disorders. Search terms for other anxiety disorders (e.g., specific phobias, panic disorder, obsessive–compulsive disorder) were not included as they reflect specific anxiety disorders.

These terms were entered into the PubMed, PsycINFO, Embase, and MEDLINE databases. Additional articles were identified through hand-search of previous relevant systematic reviews [15, 18, 19, 24–27]. Studies were eligible for inclusion if:

1. The study sample consisted of students registered in Higher Education institutions (e.g. university, college), and were exclusively undergraduate students; or a mixed sample (i.e. undergraduates and postgraduates) with findings reported separately for undergraduates.
2. The study design allowed for observation of point prevalence of non-specific anxiety (e.g. cross-sectional studies, longitudinal studies) in the studied population.
3. The study’s aim was to establish prevalence of non-specific anxiety.
4. The study used a validated outcome measure or diagnostic interview to assess general non-specific anxiety, and the outcome measure has validated cut-offs indicating different severity threshold(s) of non-specific anxiety.
5. The study reported a prevalence rate for non-specific anxiety.
6. The study reported a response rate.

Studies were excluded from this review if: 1) they were trials or intervention studies; 2) if students were undertaking secondary degrees (i.e. students had completed an undergraduate degree prior to entry to second degree); or 3) the study sample was a sub-group of the undergraduate student community.

### Data extraction

**Fig. 1:**
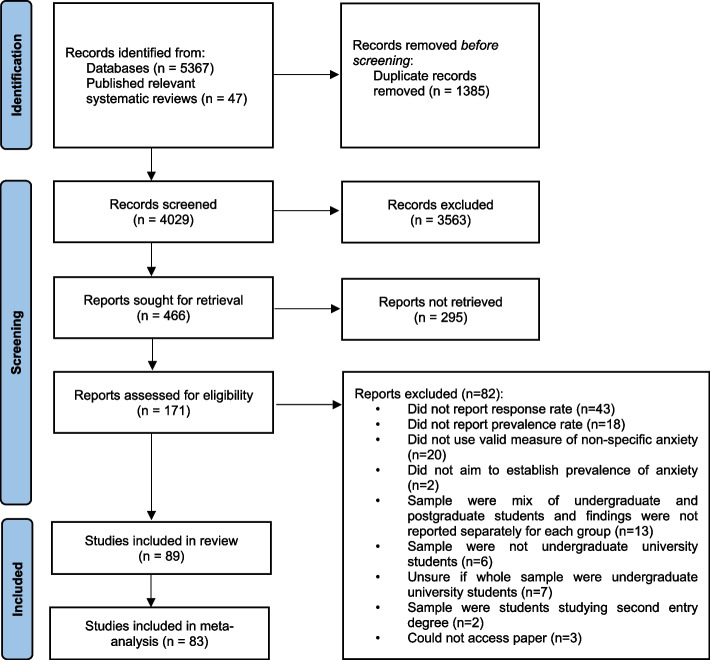
Flow diagram showing the study identification process

Two authors (EBD, IA) led the search and screening process, with disagreements resolved through discussion, with fourth author (CG) if necessary. The search results from each of the four databases and the additional 47 citations identified through previous relevant systematic reviews [15, 18, 19, 24–27] were exported to EndNote X8 [28]. All citations were collapsed together and duplicates were removed. These citations were then exported to Microsoft Excel [29], where the screening process was conducted (Fig. 1). For studies meeting the inclusion criteria, information regarding study design, sample, study location, outcome measures, estimated prevalence of non-specific anxiety and secondary analyses were extracted by EBD and IA into a Microsoft Excel spreadsheet. The World Bank classification list [30] was used to categorize the gross national income level of the countries in the included studies.

### Quality assessment of included studies

The included studies were analysed using a quality assessment instrument developed by Parker and colleagues [31] for epidemiological studies, as adapted further by Ibrahim et al. [25] in their systematic review of depression prevalence in university students. We selected this quality assessment tool as the fourth author (CG) was co-author on this previous systematic review [25] and so had expertise in using this instrument and aided us in comparing findings across the included studies. Quality assessment was conducted by EBD and IA. Using this instrument, studies were judged on presence of the following criteria:

1. The target population was defined clearly;
2. Complete, random, or consecutive recruitment was used to recruit participants;
3. The sample size was ≥300;
4. The response rate was ≥70%;
5. The sample was representative of the population being studied;
6. The outcome measure was a validated measure of non-specific anxiety, with validated cut-offs for classifying severity level(s) of non-specific anxiety; and,
7. The confidence intervals (CI) or standard error (SE) were reported for prevalence.

### Data analysis and planned analyses

The total sample size, reported prevalence for non-specific anxiety, and prevalence by severity cut-off threshold, were extracted from the included studies; studies which reported percentages only were transformed into numerical data for inclusion in meta-analysis. For studies which reported prevalence by categorical threshold (as defined by each outcome measure) but did not define an overall prevalence, the decision was taken to consider anxiety prevalence for those screening at *moderate and above* non-specific anxiety, as symptoms at this threshold are considered ‘caseness’ (i.e. likely to meet diagnostic criteria for an anxiety disorder) on several validated outcome measures (e.g. GAD-7), and symptoms at this severity are likely to be linked with functional impairment [32]. For longitudinal studies reporting multiple time points, the baseline prevalence was used for meta-analysis. We elected to use the baseline data as this most often reflected the largest sample size of any data collection timepoint. The baseline data is also likely to be the least impacted by demand characteristics or selection bias.

The primary meta-analysis performed was a pooled estimated prevalence calculated through pooling the reported prevalence in each included study. Studies which used self-report outcome measures for generalised non-specific anxiety were included in meta-analysis. Studies which used diagnostic interviews were not included in the meta-analysis given the difference in assessment and timeframe for assessing non-specific anxiety symptomology and were instead synthesised as a narrative review.

As studies can vary in their cut-offs for defining prevalence of non-specific anxiety and vary in reporting data for each cut-off, using the approach taken by Li et al.  [33], we performed secondary calculated pooled prevalence estimates at three severity levels: 1) *mild anxiety*, calculating the pooled prevalence of undergraduate students scoring at or above the cut-off for mild anxiety symptomology; 2) *Moderate anxiety*, calculating the pooled prevalence of undergraduate students scoring at or above the cut-off for moderate anxiety symptomology; and 3) *Severe anxiety*, the pooled prevalence of undergraduate students scoring at or above the cut-off for severe anxiety symptomology.

Meta-analysis of prevalence were made using the *metaprop* function in Stata (version 16.0; Stata Corporation, College Station, Texas, USA): this function uses the Freeman-Tukey double arcsine to transform prevalence estimates [34]. Results were expressed as estimated pooled prevalence of generalised anxiety calculated with 95% confidence intervals (CI). Random effect models were used to accommodate for study heterogeneity as these provide more equal weighting across studies [35] and are considered appropriate for reviews of prevalence [36]. The I^2^ statistic was used to evaluate study heterogeneity: values above 25%, 50% and 75% are considered low, moderate and high values of heterogeneity respectively [37].

For the primary meta-analysis, subgroup analyses were performed reflecting the outcome measures used to assess non-specific anxiety, course of study, and location of study as categorised using World Bank income classification (low vs. lower-middle vs. upper-middle vs. high) [30]. An additional subgroup analysis not included in the original registered protocol explored pooled prevalence by time—whether the study was conducted before or during the COVID-19 pandemic – as this global pandemic could have potentially impacted on university students’ mental health [38].

To determine the associations between sociodemographic characteristics and anxiety prevalence we extracted all findings testing such associations from the included papers. The sociodemographic variables examined were determined by previous student mental health research identifying sociodemographic factors associated with mental health outcomes in this population [25, 39]. This data was synthesised using the same narrative approach that we used for the prevalence data collected using diagnostic interviews.Publication bias of included studies was assessed through conducting an Egger test and using a Doi plot, which was quantified through using the Luis Furuya-Kanamori (LFK) index [40]. The LFK index categorises the symmetry of the funnel plot: scores <1 indicate no asymmetry; scores ≥1 to <2 indicate minor asymmetry, and scores ≥2 indicate major asymmetry.

## Results

### Study selection

After removal of duplicates, 4029 citations were retrieved from the database search and hand-search of published relevant systematic reviews. Of these a total of 89 publications—representing 86 distinct studies/samples, and totalling approx. 130,090 undergraduate students from 181 individual Higher Education Institutions (HEIs)—met eligibility criteria and were included in this review (Fig. 1; Table 1).

**Table 1:** Summary of samples and prevalence of included studies (*N* = 89)

Citation	Country	Sample								Outcome measure	Cut-off score	Overall prevalence
		**Sample size**	**Response rate**	**Age range**	**Mean age (SD)**	**Gender (% Female)**	**Course or Faculty of study**	**Year of course**	**Number of HEIs**			
Al-Bahhawi et al. (2018) [41]	Saudi Arabia	642	90.2%	NR	22.14 (1.7)	48.9%	Health sciences Other sciences Non-science courses	NR	1	DASS-21-A	≥ 10 (moderate or above)	65.7%
Abdallah & Gabr (2014) [42]	Egypt	379	90.0%	17–19 yrs	18.02 (0.26)	48.0%	Medicine	Yr 1	1	DASS-21-A	NR	78.4%
Abdel Wahed et al. (2017) [43]	Egypt	442	88.4%	17–22 yrs	20.15 (2.9)	61.1%	Medicine	Yrs 1–4	1	DASS-21-A	≥ 8 (mild and above)	64.3%
Abeetha et al. (2018) [44]	India	323	100.0%	18–22 yrs	NR	50.5%	Medicine Engineering Arts	NR	1	GAD-7	≥ 10 (moderate and above)	19.0%
Aboalshamat et al. (2015) [45]	Saudi Arabia	422	64.5%	NR	NR	53.3%	Medicine Dentistry	Yrs 2–3	1	DASS-21-A	≥ 8 (mild and above)	66.4%
Al-Khani et al. (2019) [46]	Saudi Arabia	206: 95 included in analysis	46.0%	18–28 yrs	20.8 (1.95)	24.0%	Medicine	Yrs 1–5	1	DASS-21-A	NR	28.3%
Almhdawi et al. (2018) [47]	Jordan	838	80.7%	18–41 yrs	21.3 (1.8)	77.3%	Allied health professions courses	Yrs 3–4	1	DASS-21-A	≥ 7	65.3%
Al-Shamlan et al. (2020) [48]	Saudi Arabia	523	71.1%	NR	22.4 (0.96)	54.7%	Medicine	Yrs 4–6	1	GAD-7	≥ 10 (moderate and above)	31.7%
Alvi et al. (2010) [49]	Pakistan	279	71.0%	NR	21.4 (1.41)	72.4%	Medicine	Yrs 2–5	1	BAI	≥ 8 (mild and above)	47.7%
Amir Hamzah et al. (2019) [50]	Malaysia	1631: 1602 included in analysis	53.0%	18–25	19.5 (0.7)	72.8%	NR	Yr 1	1	DASS-21-A	≥ 8 (mild and above)	60.4%
Asif et al. (2020) [51]	Pakistan	500	100.0%	18–24 yrs	NR	49.6%	NR	NR	3	DASS-21-A	≥ 8 (mild and above)	88.4%
Auerbach et al. (2016) [52]	Australia, Belgium, Germany, Mexico, UK (Northern Ireland), South Africa, Spain, USA	14,371: 13,984 included in analysis	45.5%	NR	NR	NR	NR	Yr 1	19	CIDI-SC diagnostic interview	n/a	Lifetime: 18.6% 12 month: 16.7%
Awadalla et al. (2020) [11]	UAE	404	80.8%	17–41 yrs	19.64 (2.75)	72.2%	Business Humanities and Social sciences Technological Innovation Communication and Medical Sciences Natural health sciences	Yrs 2–4	1	GAD-7	≥ 10 (moderate and above)	22.3%
Azad et al. (2017) [53]	Pakistan	150	30.0%	17–26 yrs	20.6 (0.8)	76.4%	Medicine	Yrs 1–5	1	BAI	≥ 22 (moderate or above)	13.2%
Azim et al. (2019) [54]	Pakistan	188	70.0%	18–25 yrs	21.4 (2.2)	53.0%	Medicine	Yrs 1–5	1	DASS-21-A	≥ 8 (mild and above)	72.3%
Ballester et al. (2020)^a^[55]	Spain	2118	19.0%	NR	18.8 (1.4)	72.5%	Arts and Humanities Engineering and Architecture Health Sciences Science Social and Legal Sciences	Yr 1	5	CIDI-SC diagnostic interview	n/a	Lifetime: 19.3% 12 month: 16%
Bantjes et al. (2019)^a^[56]	South Africa	1407	9.7%	NR	NR	55.2%	NR	Yr 1	2	CIDI-SC diagnostic interview	n/a	Lifetime: 22.6% 12 month: 20.8%
Bassols et al. (2014) [57]	Brazil	232	67.4%	NR	23.1 (3.2)	49.6%	Medicine	Yrs 1 and 6	1	BAI	≥ 10	18.5%
Basudan et al. (2017) [58]	Saudi Arabia	277: 247 included in analysis	95.8%	NR	NR	45.7%	Dentistry	Yrs 2–5	1	DASS-21-A	≥ 8 (mild and above)	66.8%
Baykan et al. (2012) [59]	Turkey	193	86.9%	23–31 yrs	24.5 (1.5)	44.6%	Medicine	Final year	1	DASS-42-A	≥ 8 (mild and above)	50.3%
Borst et al. (2015) [60]	Netherlands	951	43.0%	17–40	23.0 (2.6)	71.0%	Medicine	Yrs 1–6	2	BSI-ANG	> 0.41	28.0%
Bunevicius et al. (2008) [61]	Lithuania	411	92.5%	NR	21.0 (1.0)	81.7%	Medicine Humanities	NR	2	HADS-A	≥ 8 (borderline abnormal and above)	44.5%
Cheng et al. (2020) [62]	China	645	88.0%	NR	NR	55.2%	Science and Technology Literature and History Medicine	NR	3	SAS	≥ 50	25.7%
Chernomas et al. (2013) [63]	Canada	437	49.5%	NR	NR	89.0%	Nursing	Yrs 1–3	1	DASS-42-A	≥ 10 (moderate and above)	31.0%
Cheung et al. (2016) [64]	Hong Kong, China SAR	661	52.6%	18–30 yrs	NR	72.5%	Nursing	Yrs 1–4	1	DASS-21-A	≥ 10 (moderate and above)	39.9%
Cheung et al. (2020) [65]	Hong Kong, China SAR	9479	56.5%	NR	18.9 (1.5)	52.3%	Applied Science Business Construction and Environment Health and Social Science Engineering Humanities Design Hotel and Tourism Management	Yrs 1–4	1	DASS-21-A	≥ 10 (moderate and above)	29.1%
Coker et al. (2018) [66]	Nigeria	240	74.0%	25–34 yrs	25.0 (4.5)	50.0%	Medicine	Yrs 2–6	1	DASS-21-A	≥ 8	9.5%
Dalky & Gharaibeh (2019) [67]	Jordan	600	98.3%	NR	NR	64.7%	NR	NR	3	DASS-21-A	≥ 10 (moderate and above)	76.5%
Delara et al. (2015) [68]	Iran	171	93.4%	18–37 yrs	21.6 (3.3)	79.5%	Health Studies	Yrs 1–4	1	SCL-90-A	NR	35.7%
Eisenberg et al. (2007)^b^[69]	USA	1181	56.6%	NR	NR	52.8%	NR	NR	1	PHQ-A	NR	2.9%
El-Gilany et al. (2019) [70]	Egypt	900	81.9%	NR	NR	53.2%	Medicine	Yrs 1–6	1	SCL-90-A	NR	12.9%
Eller et al. (2006) [71]	Estonia	413	80.2%	19–33 yrs	21.3 (2.5)	76.9%	Medicine	Yrs 1–6	1	EST-Q	≥ 12	21.9%
El-Matury et al. (2018) [72]	Indonesia	499	80.2%	NR	NR	59.7%	Health Sciences Social Science and Humanities Science and Engineering	NR	1	DASS-21-A	NR	71.5%
Fawzy & Hamed (2017) [73]	Egypt	700	100.0%	18–25 yrs	21.22 (1.62)	64.5%	Medicine	Yrs 1–6	1	DASS-21-A	≥ 8 (mild and above)	73.0%
Fernandes et al. (2018) [74]	Brazil	205	73.0%	18–50	21.8 (3.8)	81.5%	Nursing	NR	1	BAI	≥ 8 (mild and above)	62.9%
Francis et al. (2019) [75]	Malaysia	622	89.2%	NR	21.18 (1.53)	64.8%	Medicine	Yrs 1–5	1	HADS-A	≥ 8 (borderline abnormal and above)	39.7%
Fortney et al. (2016) [76]	USA	765	31.3%	NR	NR	66.9%	NR	Yrs 1–2	11	GAD-7	≥ 10 (moderate and above)	17.6%
Gaspersz et al. (2012) [77]	Netherlands	1180	52.0%	NR	NR	NR	Medicine	Yrs 1–6	1	BSI-ANG	> 0.41	27.5%
Ge et al. (2020) [78]	China	2009	80.3%	NR	NR	51.0%	NR	Yrs 1–4	1	GAD-7	≥ 7	12.5%
Islam et al. (2020) [79]	Bangladesh	440: 400 included in analysis	88.0%	18–23 years	19.66 (0.88)	47.8%	Arts and Humanities Mathematical and Physical Sciences Social Sciences Biological Science Business Studies	Yr 1	1	GAD-7	≥ 10 (moderate and above)	61.0%
Junaid et al. (2020) [80]	Saudi Arabia	247	90.0%	NR	NR	31.2%	Medicine	Yrs 2–6	1	BAI	≥ 16 (moderate and above)	39.3%
Karaoglu & Seker (2010) [81]	Turkey	290	82.8%	NR	19.0 (1.3)	44.1%	Medicine	Yrs 1–2	1	HADS-A	≥ 10	20.3%
Kebede et al. (2020) [82]	Ethiopia	273	98.5%	NR	NR	39.4%	Medicine	Yrs 1–6	1	HADS-A	≥ 8 (borderline abnormal and above)	30.1%
Knipe et al. (2018) [83]	UK (England)	1139	55.6%	NR	NR	76.4%	Medicine Dentistry Veterinary Sciences	NR	1	GAD-7	≥ 10 (moderate and above)	27.3%
Kou et al. (2012) [84]	China	1843	90.1%	17–28	21.3 (1.6)	32.6%	Arts and Social Sciences Sciences Agriculture Engineering Medicine	Yrs 1–4	1	WHO-CIDI diagnostic interview	n/a	Lifetime: 0.5% 12 month: 0.3% 30 day: 0.1%
Kulsoom & Afsar (2015) [85]	Saudi Arabia	442	76.8%	NR	NR	38.0%	Medicine	Yrs 1–5	1	DASS-21-A	≥ 10 (moderate and above)	63% pre-examination 47% post-examination
Kumar et al. (2019) [86]	Pakistan	312	69.3%	NR	22.74 (1.52)	84.6%	Medicine	Final year	2	DASS-21-A	≥ 10 (moderate and above)	73.0%
Kunwar et al. (2016) [87]	Nepal	538	89.6%	NR	NR	52.0%	Medicine	Yrs 1–5	2	DASS-42-A	NR	41.1%
Liu et al. (1997) [88]	China	537	89.5%	NR	18.2 (1.8)	69.0%	Medicine	Yrs 1–3	1	SAS	≥ 50	12.5%
Lun et al. (2018) [89]	Hong Kong, China SAR	1119: 1101 included in analysis	81.1%	18–29	19.81 (1.48)	61.9%	NR	Yrs 1–5	8	GAD-7	≥ 5 (mild and above)	54.4%
Mahroon et al. (2018) [90]	Bahrain	307	87.7%	NR	NR	63.2%	Medicine	Yrs 1–5	1	BAI	≥ 8 (mild and above)	51.5%
Marthoenis et al. (2018) [91]	Indonesia	266	82.0%	NR	19.7 (1.8)	51.8%	NR	NR	2	GAD-7	≥ 8	27.4%
Milić et al. (2019) [92]	Croatia	562: 538 included in analysis	72.2%	20–31 yrs	NR	69.2%	Medicine Nursing	NR	1	GAD-7	≥ 5 (mild and above)	56.8%
Moutinho et al. (2017) [93]	Brazil	761	75.4%	NR	22.1 (3.3)	55.8%	Medicine	Yrs 1–6	1	DASS-21-A	NR	37.2%
Moutinho et al. (2019) [94]	Brazil	312	54.2%	NR	21.0 (2.6)	64.1%	Medicine	Yrs 1–4	1	DASS-21-A	≥ 7 (mild and above)	30.1%
Mundia (2010)^b^[95]	Brunei	68	95.9%	NR	23.6 (6.8)	NR	Education	NR	1	DASS-42-A	≥ 10 (moderate and above)	64.7%
Nahm et al. (2020) [96]	South Korea	1071: 1063 included in analysis	32.5%	NR	NR	48.0%	Veterinary Medicine	Yrs 1–6	10	DASS-21-A	≥ 15 (severe and above)	35.8%
Nakhostin-Ansari et al. (2020) [97]	Iran	323	64.6%	NR	23.73 (1.62)	52.3%	Medicine	All years	1	BAI	≥ 10 (mild and above)	38.1%
Naz et al. (2017) [98]	Pakistan	129	84.9%	17–21 yrs	19.0 (NR)	68.0%	Dentistry	Yrs 1–2	1	DASS-21-A	≥ 8 (mild and above)	41.9%
Nimkuntod et al. (2016) [99]	Thailand	213	92.6%	NR	19.51 (2.09)	56.8%	Medicine	Yrs 1–3	1	DASS-21-A	≥ 8 (mild and above)	25.8%
Paudel et al. (2020) [100]	Nepal	618	90.7%	18–29 yrs	20.39 (1.57)	62.3%	Engineering Management Law Natural Sciences Health Sciences Education Humanities	NR	NR	DASS-21-A	≥ 8 (mild and above)	46.9%
Rab et al. (2008) [101]	Pakistan	87	20.0%	18–23 yrs	20.7 (1.9)	100.0%	Medicine	Yrs 1–5	1	HADS-A	NR	43.7%
Ramón-Arbués et al. (2020) [102]	Spain	1074	80.0%	NR	21.73 (5.12)	71.0%	Health Sciences Communications Architecture and Technology	NR	1	DASS-21-A	≥ 8 (mild and above)	23.6%
Renteria et al. (2020)^a^[103]	Mexico	7874	79.3%	NR	NR	53.1%	NR	Yr 1	9	CIDI-SC diagnostic interview	n/a	12 month: 11.6%
Saeed et al. (2018) [104]	Pakistan	404	53.6%	NR	NR	52.2%	Pharmacy 'Non-pharmacy'	NR	4	DASS-42-A	≥ 8 (mild and above)	58.2%
Sahoo & Khess (2010) [105]	India	405	81.0%	NR	19.3 (2.8)	0.0%	NR	Yrs 1–2	5	DASS-21-A MINI diagnostic interview	DASS-21-A: ≥ 8 (mild and above) MINI: n/a	DASS-21-A: 24.4% MINI: 19.0%
Salem et al. (2016) [106]	Egypt	300	94.0%	NR	NR	55.7%	Pharmacy Arts	Pharmacy: Yrs 2 + 5 Arts: Yrs 1 + 4	1	DASS-21-A	≥ 8 (mild and above)	45.0%
Samaranayake et al. (2014) [107]	New Zealand	1291	66.8%	16 -38 yrs	NR	63.9%	Medicine Health sciences Nursing Law Engineering Architecture	NR	1	GAD-7	≥ 8	19.7%
Samson (2019) [108]	Nepal	680	63.4%	NR	20.29 (1.65)	NR	Nursing	Yrs 1–4	9	DASS-21-A	≥ 10 (moderate and above)	72.9%
Savitsky et al. (2020) [109]	Israel	215	88.0%	NR	Yr 1: 23.4 (2.8) Yr 2: 25.1 (2.3) Yr 3: 26.1 (3.0) Yr 4: 27.9 (3.4)	87.5%	Nursing	Yrs 1–4	1	GAD-7	≥ 10 (moderate and above)	42.8%
Serra et al. (2015) [110]	Brazil	657	97.9%	NR	22.7 (NR)	61.2%	Medicine	Yrs 1–6	1	BAI	≥ 11 (mild and above)	21.5%
Shawahna et al. (2020) [111]	State of Palestine (West Bank)	286	67.3%	NR	NR	60.8%	Medicine	Yrs 1–6	1	BAI	≥ 19 (moderate and above)	46.8%
Shen et al. (2020) [112]	China	4882	97.5%	NR	18.7 (NR)	89.0%	Medicine	NR	3	SAS	≥ 50	19.9%
Simić-Vukomanović et al. (2015) [113]	Serbia	1940	98.6%	NR	21.04 (2.23)	65.2%	Agronomy Economics Engineering Mechanical and Civil Engineering Medical Sciences Education Law Natural Sciences and Mathematics Technical Sciences Teachers Training Philology and Arts Hotel Management and Tourism	Yrs 1–6	1	BAI	≥ 8 (mild and above)	33.5%
Suarez et al. (2021) [114]	Colombia	554: 456 included in analysis	70.0%	18–25 yrs	NR	59.2%	Medicine	Yrs 1–6	1	SRQ-20-A	≥ 5	44.9%
Syed et al. (2018) [115]	Pakistan	267	100.0%	NR	19.3 (1.19)	75.3%	Physiotherapy	Yrs 1–5	NR	DASS-42-A	≥ 8 (mild and above)	68.5%
Tabalipa et al. (2015) [116]	Brazil	262	75.7%	NR	23.0 (3.3)	56.1%	Medicine	NR	1	BAI	≥ 8 (mild and above)	35.5%
Tayefi et al. (2020) [117]	Iran	560	95.0%	NR	21.2 (5.3)	55.4%	Medicine Health Sciences Allied Health Professions courses	Yr 1	1	BAI	≥ 8 (mild and above)	28.7%
Teh et al. (2015) [118]	Malaysia	397	92.3%	18–24 yrs	21.9 (2.2)	63.2%	Medicine	NR	1	DASS-21-A	≥ 10 (moderate and above)	55.4%
Torres et al. (2017) [119]	Ecuador	1110: 1092 included in analysis	99.7%	17–24 yrs	18.3 (1.1)	53.7%	Legal and Social Sciences Economic Sciences Arts and Humanities Technological Sciences Health sciences	Yr 1	1	PHQ-A	NR	0.02%
Umeh & Bangirana (2016) [120]	Uganda	387	31.5%	18–34 yrs	21.24 (2.34)	40.3%	Agriculture and Environmental Sciences Business and Management Sciences Computing and Information Sciences Education and External Studies Engineering, Design, Art and Technology Natural Sciences Humanities and Social Sciences Veterinary Medicine, Animal Resources and Biodiversity	Yr 1	1	GAD-Q-IV	≥ 5.7	28.9%
Van Der Walt et al. (2019) [121]	South Africa	473	35.2%	NR	NR	68.7%	Medicine	Yrs 1–6	1	HADS-A	≥ 8 (borderline abnormal and above)	45.9%
Van Venrooij et al. (2017) [122]	Netherlands	433	33.0%	17–33 yrs	21.2 (2.0)	75.5%	Medicine	Yrs 1–6	1	SQ-48-ANXI	NR	29.1%
Verger et al. (2010) [123]	France	1723	71.0%	18–24 yrs	19.4 (NR)	62.5%	Medicine University technology institutes Law Economics and Management Literature and Social Sciences Sciences Physical Education and Sport Sciences	Yr 1	6	CIDI-SF diagnostic interview	n/a	12 month: 2.2%
Wang et al. (2020)^b^[124]	China	39,725	80.0%	16–50 yrs	NR	54.3%	Medicine Science Engineering Literature	NR	4	SAS	≥ 50	8.1%
Wege et al. (2016) [125]	Germany	592: 590 included in analysis	73.4%	NR	21.13 (3.91)	70.0%	Medicine	Yr 1	1	GAD-7	≥ 10 (moderate and above)	1.9%
Wong et al. (2006) [32]	Hong Kong, China SAR	7915	27.5%	NR	NR	62.7%	NR	Yr 1	10	DASS-42-A	≥ 10 (moderate and above)	41.2%
Wörfel et al. (2016) [126]	Germany	1707: 1683 included in analysis	19.0%	NR	23.29 (3.72)	73.1%	Linguistics and Cultural Sciences Law, Economics and Social Sciences Engineering Human and Health Sciences Natural Sciences and Mathematics Art, Music, and Science of Art	Yrs 1–5	2	GAD-2	≥ 3	16.3%
Zeng et al. (2019) [127]	China	544	89.9%	17–24	20.2 (1.2)	97.4%	Nursing	Yrs 1–3	4	DASS-21-A	≥ 8 (mild and above)	41.7%

*NR* Not reported in paper

*Abbreviations*: *BAI* Beck Anxiety Inventory, *BSI-ANG* Brief Symptom Inventory—anxiety scale, *CIDI-SC* diagnostic interview Composite International Diagnostic Interview Screening Scales, *DASS-21-A* anxiety subscale of Depression Anxiety Stress Scales – 21 item version, *DASS-42-A* anxiety subscale of Depression Anxiety Stress Scales – 42 item version, *EST-Q* Emotional State Questionnaire, *GAD-Q-IV* Generalized Anxiety Disorder Questionnaire for DSM-IV, *GAD-2* Generalized Anxiety Disorder 2-item, *GAD-7* Generalised Anxiety Disorder scale – 7 item version, *HADS-A* anxiety subscale of Hospital Anxiety and Depression scale, *MINI* diagnostic interview—the Mini-International Neuropsychiatric Interview, *PHQ-A* Patient Health Questionnaire anxiety scale, *SAS* Zung Self-Rating Anxiety Scale, *SCL-90* anxiety subscale of the Symptom Checklist-90-Revised, *SQ-48-ANXI* anxiety subscale on Symptom Questionnaire-48, *SRQ-20-A* anxiety subscale on Self-Reporting Questionnaire 20-item version (SRQ-20) anxiety subscale, *UAE* United Arab Emirates, *UK* United Kingdom, *USA* United States of America, *WHO-CIDI* diagnostic interview World Health Organization World Mental Health Composite International Diagnostic Interview

^a^This study's data is also reported in Auerbach et al. [52]

^b^Sample was mix of undergraduates and postgraduates: table reports data for undergraduates only

### Study characteristics

The majority of studies utilised self-report measures of non-specific anxiety symptoms (*n*=83, 93.2%), and/or were cross-sectional (*n*=79, 88.7%). The remaining studies were longitudinal (*n*=4, 4.5%) or used diagnostic interviews (*n*=6, 6.7%). Sample sizes ranged from *n*=68 [95] to *n*=39,725 [124], with reported response rates ranging from 9.7% [56] to 100% [44, 51, 73, 115]. Four publications reported findings from the WHO World Mental Health Surveys International College Student Project study [52, 56, 103, 128]; this project consisted of a diagnostic interview-based survey administered to first year university students in 19 HEIs across eight countries. Four studies were conducted within the context of the COVID-19 pandemic [78, 97, 109, 124].

Using the World Bank Classification, students were recruited from a mix of high income (*n*=39), upper middle income (*n*=29), and lower middle income (*n*=22) countries, with only two studies conducted in low income countries [82, 120]. The majority of included studies were conducted in Asia (*n*=34) and the Middle East (*n*=21): nine were in Pakistan [49, 51, 53, 54, 86, 98, 101, 104, 115], seven in Saudi Arabia [41, 45, 46, 48, 58, 80, 85], seven in the People's Republic of China [62, 78, 84, 88, 112, 124, 127] and five in Egypt [42, 43, 70, 73, 106].

Thirty-eight studies reported their sample’s age range, which largely reflected a young adult age range (18–24 years). The overall age ranges ranged from 16 years [107, 124] to 50 years [74]. The 56 studies reporting samples’ mean age ranged from 18.02 years [42] to 25.0 years [66, 109]; the overall mean age from these 56 studies was calculated as 21.07 years.

Eighty-five studies reported their sample’s gender balance: two studies consisted of solely male students [105] and female students [101]. Of the remaining 83 studies, *n*=66 had a greater proportion of female students ranging from 51.8% [91] to 97.4% [127]. Over a third (*n*=38) of included studies focused solely on undergraduate medicine students, with *n*=26 sampling students across a range of undergraduate courses. Finally, *n*=39 studies recruited students from all years of study, with *n*=13 studies focusing on first years only (see Table 1).

### Outcome measures used to assess generalised anxiety

In total, 83 studies used 13 different self-report outcome measures to assess non-specific anxiety: the most commonly used outcome measure was the anxiety subscale on the Depression Anxiety Stress Scales (DASS) [129], either in its full (DASS-42-A; *n*=7) or shortened (DASS-21-A; *n*=30) format. Other commonly used outcome measures included the Generalised Anxiety Disorder scale (GAD-7) [130] (*n*=13), the Beck Anxiety Inventory (BAI) [131] (*n*=12), the anxiety subscale of the Hospital Anxiety and Depression Scale (HADS) [132] (*n*=6), and the Zung Self-Rating Anxiety Scale (SAS) [133] (*n*=4).

### Anxiety prevalence

#### Findings using studies’ own definitions

**Fig. 2:**
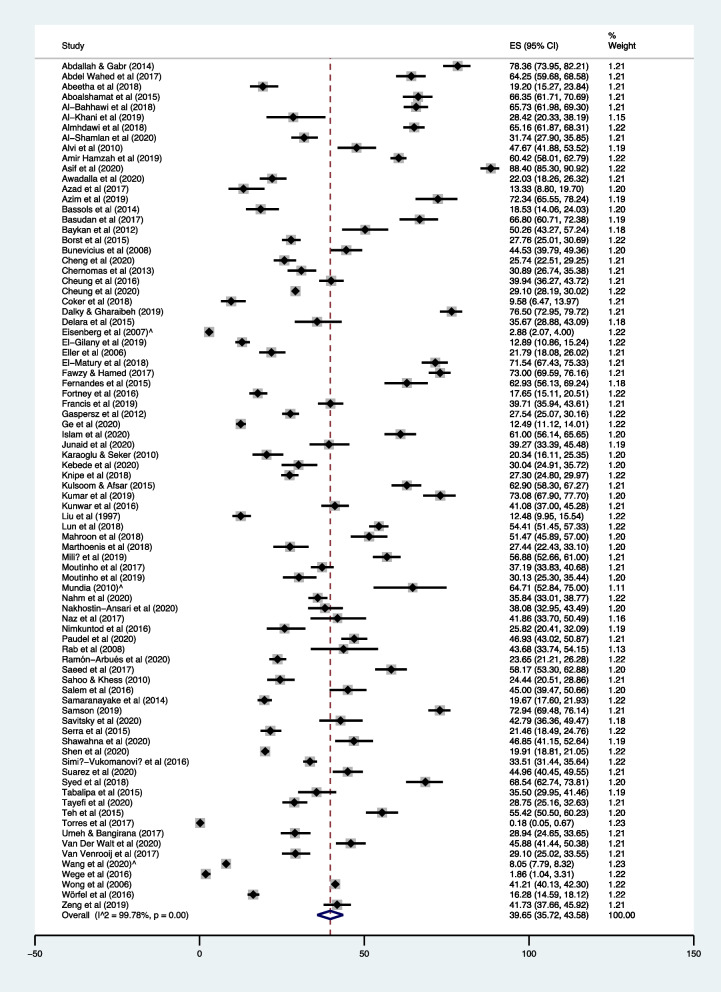
Forest plot showing prevalence of non-specific anxiety in undergraduate university students (*n* = 83 studies)

The 83 studies utilizing self-report anxiety outcome measures used a variety of definitions, criteria, and severity thresholds to define ‘prevalence’ in their sample. The prevalence reported across papers therefore reflect a broad range of values. The two studies using the brief PHQ-A reported the lowest prevalence at 0.02% [119] and 2.9% [69] screening for non-specific anxiety, while Wege et al. [125] reported 1.9% of their sample as screening for anxiety using the GAD-7. At the other end, the highest prevalence for non-specific anxiety were 78.4% [42] and 88.4% [51]: noticeably, both studies used the DASS *mild and above* cut-off. The 83 studies were included in meta-analysis using these self-defined prevalence – this resulted in a pooled prevalence of 39.65% (95% CI: 35.72%—43.58%) for non-specific anxiety, with substantial heterogeneity across the studies (I^2^ = 99.78%, *p*=<0.001) (Fig. 2).

Table 2 presents the results of the three sub-analyses. Sub-analysis reflecting the type of outcome measure revealed differences in pooled prevalence of non-specific anxiety: studies using the anxiety subscale of the DASS (either full or short version; *n*=37) reported the highest pooled prevalence at 52.1% (95% CI: 45.78%-58.42%), while studies using the HADS-A (*n*=6), BAI (*n* = 12) and GAD-7 (*n*=13) reported pooled prevalence of 30.27% (95% CI 20.41%-40.12%), 36.29% (95% CI 29.45%-43.12%) and 37.2% (95% CI 28.77%-45.64%) respectively.

**Table 2:** Subgroup analyses showing prevalence of non-specific anxiety symptoms (*n*=83 studies)

Sub-analysis	Studies (N)	Participants (N)	Pooled non-specific anxiety prevalence (%)	Lower 95% CI	Upper 95% CI	Heterogeneity (I^2^)
***Outcome measure***						
DASS-A^a^	37	34606	52.10%	45.78	58.42	99.33%*
GAD-7	13	9564	30.27%	20.41	40.12	99.47%*
BAI	12	5448	36.29%	29.45	43.12	96.50%*
HADS-A	6	2156	37.20%	28.77	45.64	94.04%*
SAS	4	45789	16.47%	8.25	24.7	99.41%*
BSI-ANG	2	2131	27.64%	25.74	29.54	n/a
SCL-90-A	2	1071	14.83%	12.73	16.92	n/a
PHQ-A	2	2273	0.36%	0.12	0.61	n/a
***Course of study***						
Medicine only	38	20565	37.42%	30.77	44.06	99.83%*
Mixture of courses	23	64,814	37.40%	31.95	42.86	99.33%*
Nursing only	6	2742	48.53%	34.05	63.00	98.43*
Dentistry only	2	376	58.76%	53.93	63.59	n/a
***World Bank classification***						
High income	29	34854	36.22%	29.50	42.94	99.60%*
Upper middle income	30	62369	35.95%	31.10	40.80	99.78%*
Lower middle income	22	8527	50.13%	38.30	61.95	99.42%*
Low income	2	660	29.39%	25.91	32.86	n/a
***Study conducted during COVID-19 pandemic***						
Pre-pandemic	79	66685	40.42%	35.04	45.80	99.85%*
During pandemic	4	42272	24.34%	16.18	32.50	99.78%*

^*^p=<.001

^a^This data includes studies which used anxiety subscale of DASS-21 or DASS-42

The prevalence of non-specific anxiety in medical student-only samples ranged from 1.9% [125] to 78.4% [42]. Studies which recruited medicine students only (*n*=38) reported a pooled prevalence of 37.42% (95% CI: 30.77%-44.06%), which is similar to the 23 studies which recruited students from a range of courses (pooled prevalence 37.40%; 95% CI 31.95%-42.86%).

When prevalence were compared by World Bank income classification, the highest pooled prevalence were found in studies conducted in lower middle income countries (*n*=22; 50.13%; 95% CI 38.30%-61.95%), with pooled prevalence being similar for high income (*n* = 29; 36.22%, 95% CI 29.50%-42.94%) and upper middle income countries (*n*=30; 35.95%, 95% CI 31.10%-40.80%).

Finally, an additional sub-analysis was undertaken to separately analyse studies conducted before and during the COVID-19 pandemic. Studies conducted before the COVID-19 pandemic resulted in a pooled prevalence of 40.42% (*n*=79; 95% CI 35.04%-45.80%), while the four studies conducted mid-pandemic reported a pooled prevalence of 24.34% (*n*=4; 95% CI 16.18%-32.50%).

#### Findings by severity threshold

The above findings are based on papers’ own definitions of anxiety prevalence, which were variable. From the 83 studies, 65 studies provided a breakdown of *mild, moderate, and severe* threshold prevalence that were usable in at least one of these secondary analyses (see Appendix A, Additional File 1). Taking data from studies reporting cases meeting the *mild and above* threshold (*n*=56), the *moderate or above* threshold (*n*=52), and *severe or above* threshold (*n*=48), the pooled estimates for mild and above non-specific anxiety was 49.94% (95% CI 41.54—58.033; I^2^ 99.84% *p*=<0.001); moderate and above non-specific anxiety was 35.64% (95% CI 29.51–41.76; I^2^ 99.63% *p*=<0.001) and severe non-specific anxiety was 20.31% (95% CI 16.60–24.02; I^2^ 99.41% *p*=<0.001).

#### Anxiety prevalence over time

Pearson’s correlation test found no statistically significant association between year of publication and reported non-specific anxiety prevalence (83 studies; *r* = 0.17, *p*=0.15).

### Narrative synthesis

#### Findings from studies using diagnostic interviews

Six publications – reflecting three separate cross-sectional studies—all used the full or screening version of the WHO Composite International Diagnostic Interview (CIDI/CIDI-SC) diagnostic interview to identify lifetime and 12-month prevalence of anxiety in undergraduate university students. Kou et al. [84] report findings from a large Chinese university student cohort (*n* = 1843), finding a 0.5% lifetime prevalence, 0.3% 12-month prevalence, and 0.1% 30-day prevalence. Using the short form version of the WHO-CIDI, Verger et al. [123] report a 2.2% 12-month prevalence in *n* = 1723 first year French undergraduates. The other four publications reflect data from the WHO World Mental Health Surveys International College Student Project: this international study used the CIDI-SC in 19 universities across eight countries to assess for diagnoses aligning with ICD and DSM-IV criteria. Auerbach et al. [52] report the overall findings from full-time first year undergraduate students (*n*=13,984), finding a 12-month prevalence of 16.7% and lifetime prevalence of 18.6%. The other three studies focus on country-specific data from the same project: Ballester et al. [55] reports a 12-month prevalence of 19.3% and 12-month prevalence of 16.0% in the Spanish student cohort. A 12-month prevalence of 11.6% was reported in a large sample of Mexican students (*n* = 7874) [103], while Bantjes et al. [56] reported a 12-month prevalence of 20.8% and lifetime prevalence of 22.6% in *n*=1407 South African students.

In addition, one cross-sectional study administered both the DASS-21 self-report measure and a diagnostic interview (Mini International Neuropsychiatric Interview/‘MINI’) [105]: the prevalence of non-specific symptoms (classified as ‘mild or above’) was reported as 24.4%, whereas prevalence of anxiety disorder on the MINI was 19%.

#### Associations between anxiety prevalence and socio-demographic factors

Fifty-eight (65.2%) studies reported findings exploring statistical associations between non-specific anxiety scores and/or anxiety threshold cut-offs and at least one socio-demographic factor (see Appendix B, Additional File 1).

##### Gender

The most commonly-reported association analysed was non-specific anxiety and gender: 48 studies conducted analyses exploring gender differences between anxiety scores and/or those meeting anxiety cut-off thresholds. Eight studies (out of 17) found that female students were significantly more likely to have higher anxiety scores than males [11, 45, 47, 58, 73, 96, 102, 109]. Only one study reported males having significantly higher scores than females [67].

Likewise, 19 studies (out of 36) found that females were more likely than males to screen above the cut-off for anxiety [11, 42, 43, 48, 49, 53, 57, 80, 82, 90, 91, 97, 100, 106, 113, 116, 121, 126].

##### Age

Thirty-one studies conducted analyses exploring associations between age and anxiety scores and/or those meeting anxiety cut-off thresholds. Six (out of 10) studies found no significant relationship between age and anxiety scores [11, 88, 92, 93, 97, 109]. Three studies reported a significant negative relationship between age and anxiety score [71, 73, 120], with one reporting a significant positive relationship [65].

This trend was also found in sixteen (out of 22) studies finding no association between age and screening above anxiety cut-offs [48–50, 71, 72, 79, 82, 87, 89, 91, 100, 104, 113, 116, 117, 127]. Three studies found younger age groups were significantly more likely to screen above anxiety cut-offs [42, 64, 70, 112], with two reporting a significant association but not describing directionality [43, 102], and one study reporting older age groups were more likely to screen for anxiety [106].

##### Year of study

In the *n* = 34 studies investigating year of study and anxiety, no consistent relationship between the two factors emerged. Seven (out of 13 studies) found no relationship between anxiety scores and year of study [11, 54, 58, 81, 96, 99, 109], with the other six reporting significant relationships: four were significantly negative relationships, with anxiety decreasing with year of study [71, 73, 93, 111].

Similarly, 13 (out of 23) studies found no statistically significant relationship between year of study and screening for anxiety cut-offs [64, 71, 81, 87–89, 97, 98, 101, 110, 116, 121, 122]. Findings from the other ten studies reporting significant relationships are inconsistent: five report that those in earlier years of study were more likely to screen for anxiety [49, 57, 70, 82, 90], while others found that later years were more likely to score above threshold for anxiety [48, 53, 80, 113, 126].

##### Ethnicity

Eight studies analysed associations between anxiety scores and/or meeting cut-off thresholds and students’ ethnicity or nationality, with five reporting no differences [50, 90, 91, 117, 121]. The other three studies all reported significant findings: Malay students were more likely to screen above anxiety threshold compared to Chinese and Indian students [118]; Saudi students were more likely to have higher mean anxiety scores compared to non-Saudi students [85]; and Han students were more likely to screen for anxiety compared to non-Han students [112].

##### Living arrangements

Fourteen (out of 20) studies found no significant associations between living arrangements and anxiety scores and/or severity cut-offs [48, 49, 57, 75, 85, 87, 90, 91, 97, 101, 111, 117, 118, 122]. The remaining six studies reported inconsistent findings: living on campus [73] and living off campus [70] was associated with elevated non-specific anxiety scores/screening above threshold, while living with family [42, 106] and living with non-relatives [50] was also associated with screening above cut-off threshold. Finally, Ramón-Arbués et al. [102] found significant differences between students who lived alone or with friends, compared to family, but did not describe directionality of findings.

##### Socioeconomic indicators

Twenty-two studies reported findings relating to anxiety and socioeconomic status (SES): this was measured/conceptualised in several different ways, including family income, parental education, and parental occupation. Three studies found no associations between anxiety scores and SES [54, 75, 93], with two studies reporting that higher anxiety scores were significantly associated with coming from families with lower income [81] and coming from less affluent families and lower maternal education, but found no association with paternal education [11].

Fifteen (out of 18) studies found no significant associations between SES and scoring above anxiety cut-offs [42, 43, 49, 53, 57, 70, 79, 87, 102, 106, 116–118, 121, 127]. Four studies reported an association between lower SES and increased risk: Simic-Vukomanovic et al. [113] and Karaoğlu and Seker [81] found students from poorer families were more likely to screen for anxiety, while Tayefi et al. [117] and Paudel et al. [100] found lower maternal and paternal education were respectively significantly associated with greater likelihood of screening for anxiety.

### Quality assessment

Each study was evaluated using seven criteria (Table 3); the majority were judged to meet three (*n*=29, 29%) four (*n*=22, 22%) or five (*n*=26, 26%) of these criteria. In terms of participant recruitment, 27 studies used random sampling methods, and 44 studies used convenience sampling – with 39 stating all students (or a particular subset) were invited to participate in the study. The criteria most-frequently judged as not being met was not reporting the CI or SE for overall anxiety prevalence (reported by *n*=15) and poor sample representativeness (reported by *n*=18). The Doi funnel plot showed high asymmetry, with Egger’s test suggesting significant publication bias (z = 3.41, *p*=0.001) and the LFK index also confirming high asymmetry (see Appendix C, Additional File 1).

**Table 3:** Quality assessment of included studies (*N*=89)

Citation	Was the final sample size ≥ *N* = 300?	Was the response rate ≥ 70%?	Did the authors use a validated measure of generalised anxiety, with valid cut-offs for classification of anxiety?	Was the target population defined clearly? (e.g. location of sample, any eligibility criteria)	Was there complete, random (stratified or non-stratified), or consecutive recruitment?	Were the confidence intervals (CI) or standard error (SE) reported?	Was the targeted sample representative, or the report presents evidence that the results can be generalized to the general undergraduate population? (could include weighting to restore representativeness)	Total quality score (out of 7)
Al-Bahhawi et al. (2018) [41]	Yes	Yes	Yes	Yes	Yes	No	Unsure	5
Abdallah & Gabr (2014) [42]	Yes	Yes	Yes	Yes	No	No	Unsure	4
Abdel Wahed et al. (2017) [43]	Yes	Yes	Yes	Yes	No	No	Yes	5
Abeetha et al. (2018) [44]	Yes	Yes	Yes	Yes	Unsure	No	No	4
Aboalshamat et al. (2015) [45]	Yes	No	Yes	Yes	No	No	No	3
Al-Khani et al. (2019) [46]	No	No	Yes	Yes	No	No	Unsure	2
Almhdawi et al. (2018) [47]	Yes	Yes	Yes	Yes	No	No	Unsure	4
Al-Shamlan et al. (2020) [48]	Yes	Yes	Yes	Yes	Yes	No	Unsure	5
Alvi et al. (2010) [49]	No	Yes	Yes	Yes	Unsure	No	No	3
Amir Hamzah et al. (2019) [50]	Yes	No	Yes	No	No	No	Yes	3
Asif et al. (2020) [51]	Yes	Yes	Yes	Yes	Yes	No	Unsure	5
Auerbach et al. (2016) [52]	Yes	No	Yes	Yes	No	Yes	Yes	5
Awadalla et al. (2020) [11]	Yes	Yes	Yes	Yes	Yes	Yes	Yes	7
Azad et al. (2017) [53]	No	No	Yes	Yes	Unsure	No	No	2
Azim et al. (2019) [54]	No	Yes	Yes	Yes	Unsure	No	No	3
Ballester et al. (2020)^a^[55]	Yes	No	Yes	Yes	No	Yes	Yes	5
Bantjes et al. (2019)^a^[56]	Yes	No	Yes	Yes	No	Yes	Yes	5
Bassols et al. (2014) [57]	No	No	Yes	Yes	Unsure	No	No	2
Basudan et al. (2017) [58]	No	Yes	Yes	Yes	No	No	Unsure	3
Baykan et al. (2012) [59]	No	Yes	Yes	Yes	No	No	No	3
Borst et al. (2015) [60]	Yes	No	Yes	Yes	No	No	Unsure	3
Bunevicius et al. (2008) [61]	Yes	Yes	Yes	Yes	Yes	No	Unsure	5
Cheng et al. (2020) [62]	Yes	Yes	Yes	Yes	Yes	No	No	5
Chernomas et al. (2013) [63]	Yes	No	Yes	Yes	No	No	No	3
Cheung et al. (2016) [64]	Yes	No	Yes	Yes	No	No	Unsure	3
Cheung et al. (2020) [65]	Yes	No	Yes	Yes	No	No	Yes	4
Coker et al. (2018) [66]	No	Yes	Yes	Yes	No	No	No	3
Dalky & Gharaibeh (2019) [67]	Yes	Yes	Yes	Yes	Unsure	No	No	4
Delara et al. (2015) [68]	No	Yes	Yes	Yes	No	No	No	3
Eisenberg et al. (2007)^b^[69]	Yes	No	Yes	Yes	Yes	No	Yes	5
El-Gilany et al. (2019) [70]	Yes	Yes	Yes	Yes	Yes	No	No	5
Eller et al. (2006) [71]	Yes	Yes	Yes	Yes	No	No	No	4
El-Matury et al. (2018) [72]	Yes	Yes	Yes	No	Yes	No	Unsure	4
Fawzy & Hamed (2017) [73]	Yes	Yes	Yes	Yes	Yes	Yes	No	6
Fernandes et al. (2018) [74]	No	Yes	Yes	Yes	No	No	Unsure	3
Fortney et al. (2016) [76]	Yes	No	Yes	Yes	Yes	Yes	Yes	6
Francis et al. (2019) [118]	Yes	Yes	Yes	Yes	No	No	No	4
Gaspersz et al. (2012) [77]	Yes	No	Yes	Yes	No	No	Unsure	3
Ge et al. (2020) [78]	Yes	Yes	Yes	No	No	No	No	3
Islam et al. (2020) [79]	Yes	Yes	Yes	Yes	Yes	No	No	5
Junaid et al. (2020) [80]	No	Yes	Yes	Yes	No	No	No	3
Karaoglu & Seker (2010) [81]	No	Yes	Yes	No	No	No	No	2
Kebede et al. (2020) [82]	No	Yes	Yes	Yes	Yes	Yes	Unsure	5
Knipe et al. (2018) [83]	Yes	No	Yes	Yes	No	No	Unsure	3
Kou et al. (2012) [84]	Yes	Yes	Yes	Yes	Yes	Yes	Yes	7
Kulsoom & Afsar (2015) [85]	Yes	Yes	Yes	Yes	No	No	Yes	5
Kumar et al. (2019) [86]	Yes	No	Yes	Yes	No	No	No	3
Kunwar et al. (2016) [87]	Yes	Yes	Yes	Yes	No	No	No	4
Liu et al. (1997) [88]	Yes	Yes	Yes	Yes	Yes	No	Unsure	5
Lun et al. (2018) [89]	Yes	Yes	Yes	Yes	No	Yes	No	5
Mahroon et al. (2018) [90]	Yes	Yes	Yes	Yes	No	No	No	4
Marthoenis et al. (2018) [91]	No	Yes	Yes	Yes	Yes	No	Unsure	4
Milić et al. (2019) [92]	Yes	Yes	Yes	Yes	Unsure	No	Yes	5
Moutinho et al. (2017) [93]	Yes	Yes	Yes	Yes	No	No	No	4
Moutinho et al. (2019) [94]	Yes	No	Yes	Yes	Unsure	No	No	3
Mundia (2010)^b^[95]	No	Yes	Yes	Yes	No	No	No	3
Nahm et al. (2020) [96]	Yes	No	Yes	Yes	Unsure	No	Unsure	3
Nakhostin-Ansari et al. (2020) [97]	Yes	No	Yes	Yes	Yes	No	No	4
Naz et al. (2017) [98]	No	Yes	Yes	Yes	No	No	No	3
Nimkuntod et al. (2016) [99]	No	Yes	Yes	Yes	Yes	No	Unsure	4
Paudel et al. (2020) [100]	Yes	Yes	Yes	Yes	Yes	No	No	5
Rab et al. (2008) [101]	No	No	Yes	Yes	Yes	No	No	3
Ramón-Arbués et al. (2020) [102]	Yes	Yes	Yes	Yes	Unsure	No	Yes	5
Renteria et al. (2020)^a^[103]	Yes	Yes	Yes	Yes	No	No	Yes	5
Saeed et al. (2018) [104]	Yes	No	Yes	Yes	Unsure	No	No	3
Sahoo & Khess (2010) [105]	Yes	Yes	Yes	Yes	Yes	No	Unsure	5
Salem et al. (2016) [106]	Yes	Yes	Yes	Yes	Yes	No	Unsure	5
Samaranayake et al. (2014) [107]	Yes	No	Yes	Yes	Unsure	Yes	No	4
Samson (2019) [108]	Yes	No	Yes	Yes	No	No	Unsure	3
Savitsky et al. (2020) [109]	No	Yes	Yes	Yes	No	No	Unsure	3
Serra et al. (2015) [110]	Yes	Yes	Yes	Yes	No	No	Unsure	4
Shawahna et al. (2020) [111]	No	No	Yes	Yes	Unsure	No	Unsure	2
Shen et al. (2020) [112]	Yes	Yes	Yes	Yes	No	No	No	4
Simić-Vukomanović et al. (2015) [113]	Yes	Yes	Yes	Yes	Yes	No	No	5
Suarez et al. (2021) [114]	Yes	Yes	Yes	Yes	No	No	Yes	5
Syed et al. (2018) [115]	No	Yes	Yes	Yes	No	No	No	3
Tabalipa et al. (2015) [116]	No	Yes	Yes	Yes	Yes	Yes	Yes	6
Tayefi et al. (2020) [117]	Yes	Yes	Yes	Yes	Unsure	No	Unsure	4
Teh et al. (2015) [118]	Yes	Yes	Yes	Yes	Unsure	No	No	4
Torres et al. (2017) [119]	Yes	Yes	Yes	Yes	Unsure	No	Yes	5
Umeh & Bangirana (2016) [120]	Yes	No	Yes	Yes	Yes	No	No	4
Van Der Walt et al. (2019) [121]	Yes	No	Yes	Yes	No	Yes	Unsure	4
Van Venrooij et al. (2017) [122]	Yes	No	Yes	Yes	No	No	No	3
Verger et al. (2010) [123]	Yes	Yes	Yes	Yes	Yes	Yes	Yes	7
Wang et al. (2020)^b^[124]	Yes	No	Yes	Yes	No	Yes	Yes	5
Wege et al. (2016) [125]	Yes	No	Yes	Yes	No	Yes	No	4
Wong et al. (2006) [32]	Yes	No	Yes	No	No	Yes	No	3
Wörfel et al. (2016) [126]	Yes	No	Yes	No	No	Yes	No	3
Zeng et al. (2019) [127]	Yes	No	Yes	Yes	Yes	Yes	Yes	6

*NR* Not reported in paper

^a^This study's data is also reported in Auerbach et al. [52]

^b^Sample was mix of undergraduates and postgraduates: table reports data for undergraduates only

## Discussion

We conducted the first systematic review and meta-analysis on the prevalence of non-specific anxiety amongst undergraduate university students. This review brings together the findings from 89 studies representing approx 130,090 participants published over a forty-year period, with 83 of these using self-report tools. Using each studies’ cut-off criteria, we found an overall pooled prevalence of 39.65% (95% CI: 35.72%—43.58%) for non-specific anxiety. Our secondary analyses found almost half (49.94%) screened for *mild and above* anxiety symptomology, a third for (35.64%) *moderate and above* symptomology, and a fifth (20.31%) reported *severe* levels of non-specific anxiety. There was no consistent pattern in terms of how anxiety prevalence or scores were associated with the year the study was conducted or sociodemographic variables, with the exception of gender; whereby anxiety tended to be more prevalent and severe amongst females rather than males.

The prevalence found here are akin to those found in previous reviews on rates of anxiety amongst medical students specifically. These reviews found estimated pooled prevalence between 7.0%-34.5% [14, 15, 19, 20, 27, 134, 135]. Our own secondary analysis further supports this finding as the pooled prevalence and associated confidence intervals were almost identical when looking at medicine students (37.42%, 95% CI 30.77%-44.06%) alone compared to undergraduate students generally (37.40%, 95% CI 31.95%-42.86%). Our findings question the rhetoric that medical students are particularly vulnerable to poor mental health above other students from other disciplines [136]. Instead, we can conclude that anxiety is an issue that can affect all students, irrespective of their area of study. Furthermore, through a sub-analysis we were able to calculate a pooled prevalence of 40.42% in studies conducted before the COVID-19 pandemic. These pre-pandemic rates align with a recent systematic review of 36 studies assessing anxiety prevalence in university students during the COVID-19 pandemic, reporting a pooled prevalence of 41.0% [38]. However, it is important to acknowledge the significant heterogeneity found in relation to these prevalence estimates, meaning the pooled value is not necessarily a valid reflection of the literature. Unpacking this heterogeneity was beyond the scope of this review but is an important question that needs to be addressed.

Across the main and sub-analyses within this review we consistently found evidence for a concerning level of anxiety amongst undergraduate students. Our findings compliment those of other reviews on the prevalence of common mental health problems in university students [14, 18, 25, 137]. The prevalence found in diagnostic interview studies here far exceed the lifetime prevalence found using WHO data (3.7%) [4], and students may therefore be considered a high-risk group that requires special attention and support. These results support the frequent media reports of an ongoing ‘student mental health crisis’ [6].

Compared to other epidemiological studies, the cross-sectional prevalence found here are on par with those found in the general population (e.g. Bandelow & Michaelis, 2015 [138]). Whether students are especially vulnerable to anxiety or not, the prevalence found here alarmingly suggest that more than third of students are experiencing anxiety symptoms that likely meet diagnostic thresholds and therefore require intervention (i.e. of moderate or greater severity). In light of the negative sequelae of anxiety amongst students, including impaired academic performance [10, 11] which then has implications for their future employment prospects, we assert a need to explore this issue further.

We attempted to do this in the present review by conducting secondary analyses to identify potential sociodemographic risk factors within the student population that may elevate students’ risk of anxiety. We found no consistent evidence that any of the sociodemographic variables were associated with increased anxiety symptoms, with the exception of gender – aligning with findings from a previous systematic review finding female students reported greater prevalence of depression compared to males [25]. While we are unable to comment on the rates of anxiety amongst non-binary or gender non-conforming students, we did find that studies largely reported that anxiety was more prevalent amongst females than males. In the general population, rates of anxiety disorders, irrespective of the sub-type, are more common amongst females than males [139]. This finding may be explained by the increased prevalence of anxiety-related risk factors amongst females than males. For example, stress sensitivity and hormonal changes may contribute to the increased incidence of anxiety amongst females [140]. Females may also be more likely to experience the kinds of traumatic life events that can trigger anxiety, such as sexual violence [141] or relationship difficulties [140]. It may therefore be most appropriate to target any measures related to preventing anxiety disorders to female university students.

This does not mean that male students are invulnerable to mental health problems, including anxiety disorders [67]. This finding should be considered in light of the gender biases seen in the presentation and assessment of mental health difficulties. For example, there is strong evidence of gender biases in the diagnostic assessment of several mental health difficulties—but whether such a bias exists for anxiety disorders has not been fully explored [142]. Moreover, the gender differences found here could be a product of gender norms in relation to how distress is expressed (i.e. tendency to internalise versus externalise) [143] as well as gender differences in willingness to disclose mental health difficulties [144].

The inconsistent findings concerning the relationship between non-specific anxiety prevalence and sociodemographic factors contradicts previous literature. Studies within the general population have shown that anxiety is more common amongst those who are younger [145], an ethnic majority [146], or are of a lower SES [147] – whereas we found studies that both supported and disproved these findings. Similarly, previous studies have suggested there are key ‘pinch points’ over the course of studying for a degree that are associated with increased mental health difficulties [148]; however, we did not find any coherent narrative concerning the phase of study and anxiety prevalence specifically.

The mixed findings presented here may be explained in part by cross-cultural differences. That is, we included studies from around the world and, as mental health problems are culturally bound [149], it is likely that this will result in some between-study heterogeneity in how anxiety is understood, conceptualised, and assessed. A more likely explanation for the heterogeneity in our results, other than cultural factors, are the methodological differences between studies [138]—specifically, the different measures used across studies to assess anxiety. There may be logistical issues surrounding access to validated outcome measures of non-specific anxiety; for example, the DASS is in the public domain with an accompanying publicly available website [150] suggesting this measure could be more easily accessible to researchers. The findings from the six studies using diagnostic clinical interviews reported 12-month prevalence generally lower than those found in self-report studies, ranging from 0.3% [84] to 20.8% [56]. While these studies provide more robust findings, given diagnostic interviews are the gold-standard [151], self-report measures are used widely and have been validated as a means to assessing anxiety symptoms in a less time-intensive and resourceful manner.

### Limitations

Self-report measures are a valid means of assessing anxiety symptoms, but there are multiple outcome measures available in the literature. More than 145 anxiety outcome measures have been published [152]. In the present review we pooled data from the DASS [129], GAD-7 [130], BAI [131], HADS [132], and SAS [133]. Although all these scales are measuring the same latent variable, they differ in their conceptualisation of it. For example, the DASS focusses on the physiological symptoms of anxiety (e.g., trembling, dry mouth, heart palpitations), whereas the GAD-7 primarily assesses psychological and cognitive symptoms (e.g., worrying, nervousness, irritablility). Although the pooling of data from multiple anxiety questionnaires is common practice within literature reviews, it is a questionable practice that is likely to produce biased results [153]. This limitation seems to be somewhat justified here given the findings of our sub-analysis: we found that anxiety prevalence estimates varied depending on the measure used. These differences may reflect real differences but are more likely an artefact of measurement error – this limitation has consequences for the validity of our findings. This may also reflect logistical issues around the accessibility of anxiety outcome measures and associated manuals/protocols to researchers in lower/middle income countries.

The studies included in this review may be variable in the outcome measures they used but they are largely from the same part of the world. Most of the studies were conducted in Asian countries or the Middle East. Very few of the studies included were from Western countries. This is very different from the patterns seen in other reviews on student mental health where there is a dominance of data from the United States (US) and the UK (e.g. [154]). This may be because of the inclusion/exclusion employed in the present review – specifically that we were only interested in studies that reported both prevalence and response rates, meaning we may have excluded studies due to not reporting response rates. There may be something about the way universities are set up in Asia and the Middle East that makes it more feasible to conduct population screening studies to ascertain prevalence estimates, compared to institutions based in the UK and US (e.g. size of universities or level of state involvement). Our findings therefore highlight a gap in Western literature that needs addressing but also potentially limits the extent to which we can generalise our findings to all parts of the world.

Applying the quality assessment criteria used to assess previous epidemiological studies [25, 31], overall many studies had decent sample sizes and response rates. However, the majority of papers did not include any additional information about their data; for example, few described how their sample aligned with their HEI’s sociodemographic make-up or how it compared to the wider university student population. Researchers conducting prevalence studies may wish to use published guidelines for best practice in reporting observational studies, such as the STROBE checklist [155] when reporting the findings from epidemiological observational studies in order to improve their quality and the quality of reviews thereafter.

### Implications

Even with the limitations above, our review provides strong evidence that anxiety is prevalent amongst undergraduate students. Anxiety, however, is an umbrella term that encompasses several distinct mental health disorders within diagnostic manuals [156, 157]. While there are some generic interventions that appear to be effective for anxiety disorders and common mental health problems broadly—such as antidepressants and low intensity psychological interventions [158]—there are increased treatment options available when the typology of the anxiety is known [159–161]. Further investigation is needed to specify the presentation of this non-specific anxiety so as to inform intervention recommendations and provision at universities.

The high prevalence of anxiety amongst undergraduate students suggests that there may be something about their student status that is elevating this risk beyond that seen in the general population. There may be aspects of student life, their studies or the university environment that are triggering anxiety symptoms. Universities should be safe spaces that give their students every opportunity to flourish and achieve their potential. It is therefore vital that we identify what aspects of the university experience are distressing students and seek to address these without delay. There are some suggestions within the literature as to what these factors may be; including, workload pressures, fear of failure, imposter feelings, financial difficulties, as well as poor social support and networks [162] and cultural changes in society [17]. Targeting such diverse and disparate risk factors within a single intervention is impractical. We therefore need to explore and prioritise these issues and consider ways to mitigate them.

Through a research priority setting exercise with UK university students [163], students have identified several directions for future research into student mental health: this includes exploring the effectiveness of university-based mental health services, and clinical and non-clinical interventions, and how prevalence of mental health problems differs across institutions, discipline of study and by socio-demographic characteristics. The findings from our systematic review are particularly relevant in helping answer these research priorities: for example, in identifying the effectiveness of interventions to help students’ mental health, one important factor in this is considering how we can measure mental health outcomes and different measures used to assess the same construct.

Finally, our review suggests that female students are at an elevated risk of anxiety compared to males. If this finding reflects a genuine gender difference that cannot be explained by gender biases, we must consider whether this gender difference is similar or greater than that found in the general population. Are females particularly vulnerable to the university-related risk factors causing student anxiety or are these gender differences reflective of what we see in wider society? This question highlights the need for intersectional research in this area so as to understand the interaction and cumulative effects of risk factors on poor mental health (e.g. [164, 165]).

## Conclusions

The primary aim of this review was to produce a pooled estimate of the prevalence of non-specific anxiety amongst undergraduate students. We found an overall pooled prevalence of 39.65%—a figure that exceeds those seen in epidemiological studies in the general population. Students may therefore be a high-risk group, with some suggestion here that this risk may be further elevated for female students. There is a need to understand how best to support students with anxiety, and why anxiety is increasingly common amongst this group.

## Supplementary Information

**Additional file 1: Appendix A.** Table showing prevalence by outcome measure cut-off thresholds for each included study. **Appendix B.** Table showing associations reported between anxiety scores, anxiety threshold cut-offs and sociodemographic variables in included studies. **Appendix C.** Doi funnelplot for the 83 studies included in meta-analysis. **Appendix D.** Full citation list of 89 studies included in systematic review.

**Additional file 2.** PRISMA checklist.

**Additional file 3.** Dataset showing anxiety prevalence data from each included study.

## Data Availability

All data generated or analysed during this study are included in this published article and its supplementary information files.

## Abbreviations

BAI: Beck Anxiety Inventory
CI: Confidence intervals
DASS: Depression Anxiety Stress Scales
GAD-7: Generalised Anxiety Disorder scale – 7-item version
HADS: Hospital Anxiety and Depression Scale
HEIs: Higher Education Institutions
PHQ-A: Patient Health Questionnaire anxiety scale
SAS: Zung Self-Rating Anxiety Scale
SES: Socioeconomic status
