# Supplementary material for: A systematic review and meta-analysis of studies exploring prevalence of non-specific anxiety in undergraduate university students
Source: BMC Psychiatry. 2023 Apr 11;23:240. doi: 10.1186/s12888-023-04645-8 (PMC10088287; doi:10.1186/s12888-023-04645-8)
Supplement: Supplementary file 1 — Additional file 1: Appendix A. Table showing prevalence by outcome measure cut-off thresholds for each included study. Appendix B. Table showing associations reported between anxiety scores, anxiety threshold cut-offs and sociodemographic variables in included studies. Appendix C. Doi funnelplot for the 83 studies included in meta-analysis. Appendix D. Full citation list of 89 studies included in systematic review. [file 12888_2023_4645_MOESM1_ESM.docx]

**Additional Files**

**Appendix A:** Prevalence by outcome measure cut-off thresholds for each included study (n=89)

| **Citation** | **Outcome measure** | **Reported prevalence by cut-off threshold for measure** | | | | |
| --- | --- | --- | --- | --- | --- | --- |
|  |  | **'None'/'Minimal'** | **'Mild'** | **'Moderate'** | **'Severe'** | **'Extremely severe'** |
| Al-Bahhawi et al. (2018) | DASS-21-A | 170 / 26.5% | 50 / 7.8% | 156 / 24.3% | 86 / 13.4% | 180 /28.0% |
| Abdallah & Gabr (2014) | DASS-21-A | NR | 23 / 6.1% | 165 / 43.6% | 51 / 13.5% | 140 / 36.8% |
| Abdel Wahed et al. (2017) | DASS-21-A | 158 / 35.7% | 152 / 34.4% screened for *mild or moderate* anxiety | | 132 / 29.9% screened for *severe or extremely severe* anxiety | |
| Abeetha et al. (2018) | GAD-7 | 261 / 81.1% screened for *minimal or mild* anxiety | | 62 / 19.1% screened for *moderate or severe anxiety* | | N/A |
| Aboalshamat et al. (2015) | DASS-21-A | 142 / 33.6% | 86 / 20.4% | 60 / 14.2% | 43 / 10.2% | 91 / 21.6% |
| Al-Khani et al. (2019) | DASS-21-A | NR | 3 / 3% screened for *mild or moderate* anxiety | | 24 /25.3% screened for *severe or extremely severe* anxiety | |
| Almhdawi et al. (2018) | DASS-21-A | 290 / 34.7% | 72 / 8.6% | 157 / 18.8% | 92 /11.0% | 225 / 26.9% |
| Al-Shamlan et al. (2020) | GAD-7 | 217 / 41.5% | 140 / 26.8% | 91 / 17.4% | 75 / 14.3% | N/A |
| Alvi et al. (2010) | BAI | 146 / 52.3% | 77 /27.6% | 38 / 13.6% | 18 / 6.5% | N/A |
| Amir Hamzah et al. (2019) | DASS-21-A | 634 / 39.6% | 175 / 10.9% | 456 / 28.5% | 183 / 11.4% | 154 / 9.6% |
| Asif et al. (2020) | DASS-21-A | 58 / 11.6% | 22 / 4.4% | 97 / 19.4% | 89 / 17.8% | 234 / 46.8% |
| Auerbach et al. (2018) | CIDI-SC diagnostic interview | N/A | N/A | N/A | N/A | N/A |
| Awadalla et al. (2020) | GAD-7 | 315 / 77.7% screened for *minimal or mild* anxiety | | 89 / 22.3% screened for *moderate or severe anxiety* | | N/A |
| Azad et al. (2017) | BAI | 129 / 86.1% screened for *minimal or mild* anxiety | | 20 / 13.2% screened for *moderate or severe anxiety* | | N/A |
| Azim et al. (2019) | DASS-21-A | 52 / 27.6% | 29 / 15.4% | 82 / 43.6% | 14 / 7.4% | 11 / 5.9% |
| Ballester et al. (2020)* | CIDI-SC diagnostic interview | N/A | N/A | N/A | N/A | N/A |
| Bantjes et al. (2019)* | CIDI-SC diagnostic interview | N/A | N/A | N/A | N/A | N/A |
| Bassols et al. (2014) | BAI | NR | 28 / 12.0% | 11 / 4.7% | 4 / 1.7% | N/A |
| Basudan et al. (2017) | DASS-21-A | 82 / 33.2% | 28 / 11.3% | 52 / 21.4% | 22 / 8.9% | 62 / 25.1% |
| Baykan et al. (2012) | DASS-42-A | NR | NR | NR | NR | NR |
| Borst et al. (2015) | BSI-ANG | NR | NR | NR | NR | NR |
| Bunevicius et al. (2008) | HADS-A | 228 / 55.5% | N/A | N/A | N/A | N/A |
| Cheng et al. (2020) | SAS | 479 / 74.3% | 134 / 20.7% | 32 / 5.0% | 0 / 0.0% | N/A |
| Chernomas et al. (2013) | DASS-42-A | 267 / 61% | 35 / 8% | 65 / 15% | 35 / 8% | 35 / 8% |
| Cheung et al. (2016) | DASS-21-A | 335 / 50.6% | 62 / 9.5% | 264 / 39.9% screened for *moderate, severe or extremely severe anxiety* | | |
| Cheung et al. (2020) | DASS-21-A | NR | NR | NR | NR | NR |
| Coker et al. (2018) | DASS-21-A | 217 / 90.4% | 16 / 6.6% | 3 / 1.2% | 2 / 0.8% | 2 / 0.8% |
| Dalky & Gharaibeh (2019) | DASS-21-A | 111 / 18.5% | 29 / 4.8% | 110 / 18.3% | 88 / 14.7% | 261 / 43.5% |
| Delara et al. (2015) | SCL-90-A | 110 / 64.3% | N/A | N/A | N/A | N/A |
| Eisenberg et al. (2007)^ | PHQ-A | 1147 / 97.1% | N/A | N/A | N/A | N/A |
| El-Gilany et al. (2019) | SCL-90-A | 784 / 87.1% | N/A | N/A | N/A | N/A |
| Eller et al. (2006) | EST-Q | 323 / 78.1% | N/A | N/A | N/A | N/A |
| El-Matury et al. (2018) | DASS-21-A | 24 / 4.8% | 23 / 4.6% | 44 / 8.8% | 51 /10.2% | 357 / 71.5% |
| Fawzy & Hamed (2017) | DASS-21-A | 189 / 27.0% | 113 / 16.1% | 111 / 15.9% | 87 / 12.4% | 200 / 28.6% |
| Fernandes et al. (2018) | BAI | 80 / 37.1% | 62 / 30.2% | 44 / 21.5% | 23 / 11.2% | N/A |
| Francis et al. (2019) | HADS-A | 375 / 60.3% | 121 / 19.5% | 106 / 17.0% | 20 / 3.2% | N/A |
| Fortney et al. (2016) | GAD-7 | NR | NR | 135 / 17.6% screened for *moderate or severe anxiety* | | N/A |
| Gaspersz et al. (2012) | BSI-ANG | NR | NR | NR | NR | NR |
| Ge et al. (2020) | GAD-7 | NR | NR | NR | NR | NR |
| Islam et al. (2020) | GAD-7 | 72 / 18% | 84 / 21% | 189 / 47.3% | 55 / 13.8% | N/A |
| Junaid et al. (2020) | BAI | 82 / 33% | 68 / 27% | 59 / 24% | 38 / 16% | N/A |
| Karaoglu & Seker (2010) | HADS-A | NR | NR | NR | NR | NR |
| Kebede et al. (2020) | HADS-A | N/A | N/A | N/A | N/A | N/A |
| Knipe et al. (2018) | GAD-7 | 828 / 72.7% screened for *minimal or mild* anxiety | | 174 / 15.6% | 137 / 12.0% | N/A |
| Kou et al. (2012) | WHO-CIDI diagnostic interview | N/A | N/A | N/A | N/A | N/A |
| Kulsoom & Afsar (2015) | DASS-21-A | Pre exam: 164 / 37% *none or mild* Post-exam: 234 / 53% *none or mild* | | Pre exam: 278 / 63% *moderate, severe, or extremely severe* Post-exam: 208 / 47% *moderate, severe, or extremely severe* | | |
| Kumar et al. (2019) | DASS-21-A | 60 / 19.2% | 36 / 11.5% | 12 / 3.8 % | 48 / 15.4% | 168 / 53.8% |
| Kunwar et al. (2016) | DASS-42-A | 317 / 58.9% | NR | NR | NR | NR |
| Liu et al. (1997) | SAS | NR | NR | NR | NR | N/A |
| Lun et al. (2018) | GAD-7 | 502 / 45.6% | 379 / 34.4% | 156 / 14.2% | 64 / 5.8% | N/A |
| Mahroon et al. (2018) | BAI | 149 / 48.5% | 72 / 23.5% | 57 / 18.6% | 29 / 9.4 | N/A |
| Marthoenis et al. (2018) | GAD-7 | 120 / 45.1% | 102 / 38.3% | 39 / 14.7% | 5 / 1.9% | N/A |
| Milić et al. (2019) | GAD-7 | 245 / 45.5% | 214 / 39.8% | 68 / 12.6% | 11 / 2.0% | N/A |
| Moutinho et al. (2017) | DASS-21-A | NR | NR | NR | 93 / 12.2% *severe or extremely severe* | |
| Moutinho et al. (2019) | DASS-21-A | NR | NR | NR | NR | NR |
| Mundia (2010)^ | DASS-42-A | 12 / 17.6% | 12 / 17.6% | 27 / 39.7% | 12 / 17.6% | 5 / 7.4% |
| Nahm et al. (2020) | DASS-21-A | NR | NR | NR | NR | NR |
| Nakhostin-Ansari et al. (2020) | BAI | 200 / 61.9% | 77 / 23.8% | 31 / 9.6% | 15 / 4.6% | N/A |
| Naz et al. (2017) | DASS-21-A | 75 / 58.1% | 54 / 41.9% screened *mild and above* | | | |
| Nimkuntod et al. (2016) | DASS-21-A | 158 / 74.2% | 28 / 13.1% | 20 / 9.4% | 5 / 2.3% | 2 / 0.9% |
| Paudel et al. (2020) | DASS-21-A | 328 / 53.1% | 49 / 7.9% | 126 / 20.4% | 115 / 18.6% | NR |
| Rab et al. (2008) | HADS-A | N/A | N/A | N/A | N/A | N/A |
| Ramón-Arbués et al. (2020) | DASS-21-A | 820 / 76.4% | 87 / 8.1% | 96 / 8.9% | 9 / 0.8% | 62 / 5.8% |
| Renteria et al. (2020)* | CIDI-SC diagnostic interview | N/A | N/A | N/A | N/A | N/A |
| Saeed et al. (2017) | DASS-42-A | 169 / 41.8 % | 41 / 10.1% | 66 / 16.3% | 38 / 9.4% | 90 / 22.2% |
| Sahoo & Khess (2010) | DASS-21-A | 306 / 75.6% | 22 / 5.4% | 50 / 12.3% | 6 / 1.5% | 21 / 5.2% |
| Salem et al. (2016) | DASS-21-A | 165 / 55% | 72 / 24% | 48 / 16% | 15 / 5% | NR |
| Samaranayake et al. (2014) | GAD-7 | NR | NR | NR | NR | N/A |
| Samson (2019) | DASS-21-A | 127 / 18.7% | 57 / 8.4% | 171 / 25.1% | 106 / 15.6% | 219 / 32.2% |
| Savitsky et al. (2020) | GAD-7 | 123 / 57.2% screened for *minimal or mild* anxiety | | 53 / 24.7% | 39 / 18.1% | N/A |
| Serra et al. (2015) | BAI | 516 / 79.5% | 104 / 15.8% | 29 / 4.4% | 8 / 1.2% | N/A |
| Shawahna et al. (2020) | BAI | 67 / 23.4% | 85 / 29.7% | 73 / 25.5% | 61 / 21.3% | N/A |
| Shen et al. (2020) | SAS | NR | NR | NR | NR | N/A |
| Simić-Vukomanović et al. (2015) | BAI | 1290 / 66.5% | 440 / 22.7% | 144 / 7.4% | 66 / 3.4% | N/A |
| Suarez et al. (2020) | SRQ-20-A | NR | NR | NR | NR | NR |
| Syed et al. (2018) | DASS-42-A | 84 / 31.4% | 37 / 13.9% | 64 / 24.0% | 44 / 16.5% | 38 / 14.2% |
| Tabalipa et al. (2015) | BAI | 169 / 64.5% | 65 / 24.8% | 23 / 8.8% | 5 / 1.9% | N/A |
| Tayefi et al. (2020) | BAI | 399 / 71.3% | 116 / 20.7% | 40 / 7.1% | 5 / 0.9% | N/A |
| Teh et al. (2015) | DASS-21-A | 143 / 36.0% | 34 / 8.6% | 121 / 30.5% | 40 / 10.1% | 59 / 14.9% |
| Torres et al. (2017) | PHQ-A | 1090 / 99.2% | N/A | N/A | N/A | N/A |
| Umeh & Bangirana (2017) | GAD-Q-IV | 275 / 71.1% | N/A | N/A | N/A | N/A |
| Van Der Walt et al. (2020) | HADS-A | 256 / 54.1% | N/A | N/A | N/A | N/A |
| Van Venrooij et al. (2017) | SQ-48-ANXI | 308 / 70.9% | N/A | N/A | N/A | N/A |
| Verger et al. (2010) | CIDI-SF diagnostic interview | N/A | N/A | N/A | N/A | N/A |
| Wang et al. (2020)^ | SAS | NR | NR | NR | NR | N/A |
| Wege et al. (2016) | GAD-7 | 579 / 98.1% screened for *minimal* or *mild anxiety* | | 11 / 1.9% screened for *moderate* or *severe anxiety* | | N/A |
| Wong et al. (2006) | DASS-42-A | 3698 / 46.7% | 955 / 12.1% | 1763 / 22.3% | 895 / 11.3% | 604 / 7.6% |
| Wörfel et al. (2016) | GAD-2 | 1409 / 83.7% | N/A | N/A | N/A | N/A |
| Zeng et al. (2019) | DASS-21-A | 317 / 58.3% | 186 / 34.2% screened *mild* or *moderate* | | 41 / 7.5% screened *severe* or *extremely severe* | |

* This study's data is also reported in Auerbach et al. (2016).

^Sample was mix of undergraduates and postgraduates: table reports data for undergraduates only.

NR: Not reported in paper

N/A: Outcome measure does not have this threshold(s) or is not applicable to the outcome measure.

**Abbreviations:** BAI – Beck Anxiety Inventory; BSI-ANG - Brief Symptom Inventory - anxiety scale; CIDI-SC diagnostic interview - Composite International Diagnostic Interview Screening Scales; DASS-21-A – anxiety subscale of Depression Anxiety Stress Scales – 21 item version; DASS-42-A - anxiety subscale of Depression Anxiety Stress Scales – 42 item version; EST-Q - Emotional State Questionnaire; GAD-Q-IV - Generalized Anxiety Disorder Questionnaire for DSM-IV; GAD-2 - Generalized Anxiety Disorder 2-item; GAD-7 – Generalised Anxiety Disorder scale – 7 item version; HADS-A – anxiety subscale of Hospital Anxiety and Depression scale; MINI diagnostic interview - the Mini-International Neuropsychiatric Interview; PHQ-A – Patient Health Questionnaire anxiety scale; SAS - Zung Self-Rating Anxiety Scale; SCL-90-A – anxiety subscale of the Symptom Checklist-90-Revised; SQ-48-ANXI – anxiety subscale on Symptom Questionnaire-48; SRQ-20-A – anxiety subscale on Self-Reporting Questionnaire 20-item version (SRQ-20) anxiety subscale; WHO-CIDI diagnostic interview - World Health Organization World Mental Health Composite International Diagnostic Interview

**Appendix B.** Outline of associations reported between anxiety scores, anxiety threshold cut-offs and sociodemographic variables in included studies (N=89).

| **Citation** | **Did they look at sociodemographic factors?** | **Sociodemographic variables and anxiety** | | | | | | | | | | | | | |
| --- | --- | --- | --- | --- | --- | --- | --- | --- | --- | --- | --- | --- | --- | --- | --- |
|  |  | **Gender: Anxiety Scores** | **Gender: anxiety cut-off** | **Age: anxiety scores** | **Age: anxiety cut-off** | **Course of study: anxiety scores** | **Course of study: anxiety cut-off** | **Yr of study: anxiety scores** | **Yr of study: anxiety cut-off** | **Ethnicity or nationality: anxiety scores** | **Ethnicity or nationality: anxiety cut-off** | **Living arrangements: anxiety scores** | **Living arrangements: : anxiety cut-off** | **Socioeconomic status: anxiety scores** | **Socioeconomic status: anxiety cut-off** |
| Al-Bahhawi et al. (2018) | No | NR | NR | NR | NR | NR | NR | NR | NR | NR | NR | NR | NR | NR | NR |
| Abdallah & Gabr (2018) | Yes | NR | Females sig more likely to screen above the cut-off for anxiety | NR | NR | NR | NR | NR | NR | NR | NR | NR | Students living with family sig more likely to screen above anxiety cut-off, compared to students in university housing or living with friends | NR | No sig relationship between screening for anxiety cut-off and perceived socioeconomic standard ('low', 'moderate', 'high') |
| Abdel Wahed et al. (2017) | Yes | NR | Females sig more likely to screen above the cut-off for anxiety | NR | Sig association between age and anxiety cut-offs: direction not described | NR | NR | NR | NR | NR | NR | NR | NR | NR | No sig relationship between screening for anxiety cut-off and socioeconomic standard ('very low', 'low', 'middle', 'high') |
| Abeetha et al. (2018) | No | NR | NR | NR | NR | NR | NR | NR | NR | NR | NR | NR | NR | NR | NR |
| Aboalshamat et al. (2015) | Yes | Females studying medicine had sig higher scores compared to males studying medicine or dentistry | NR | NR | NR | Females studying medicine had sig higher scores compared to males studying medicine or dentistry | NR | NR | NR | NR | NR | NR | NR | NR | NR |
| Al-Khani et al. 2019 | No | NR | NR | NR | NR | NR | NR | NR | NR | NR | NR | NR | NR | NR | NR |
| Almhdawi et al. 2018 | Yes | Females sig higher scores | No sig gender diffs | NR | NR | Radiologic Technology students had sig higher anxiety scores than other courses | NR | NR | NR | NR | NR | NR | NR | NR | NR |
| Al-Shamlan et al. (2020) | Yes | NR | Females sig more likely to screen above the cut-off for anxiety | NR | No association between age and anxiety cut-offs | NR | NR | NR | Being in 4th yr sig associated with more likely to screen for anxiety | NR | NR | NR | No sig relationship between screening for anxiety cut-off and living arrangements | NR | NR |
| Alvi et al. (2010) | Yes | NR | Females sig more likely to screen above the cut-off for anxiety | NR | No association between age and anxiety cut-offs | NR | NR | NR | Being in 2nd yr sig associated with more likely to screen for anxiety | NR | NR | NR | No sig relationship between screening for anxiety cut-off and living arrangements | NR | No sig relationship between screening for anxiety cut-off and maternal or paternal occupation |
| Amir Hamzah et al. (2019) | Yes | NR | NR | NR | No association between age and anxiety cut-offs | NR | No association between course of study and anxiety cut-offs | NR | NR | NR | No sig differences between Malay, Chinese, Indian and Other groups | NR | Students living with non-family members sig more likely to screen above anxiety cut-off, compared to students living with family | NR | NR |
| Asif et al. (2020) | No | NR | NR | NR | NR | NR | NR | NR | NR | NR | NR | NR | NR | NR | NR |
| Auerbach et al. 2018 | No | NR | NR | NR | NR | NR | NR | NR | NR | NR | NR | NR | NR | NR | NR |
| Awadalla et al. (2020) | Yes | Females sig higher scores | Females sig more likely to screen above the cut-off for anxiety | No sig relationship | NR | NR | NR | No sig relationship | NR | NR | NR | NR | NR | Higher anxiety scores sig associated with coming from less affluent families and lower maternal education  No association between anxiety scores and paternal education | NR |
| Azad et al. (2017) | Yes | NR | Females sig more likely to screen above the cut-off for anxiety | NR | NR | NR | NR | NR | Being in 5th yr sig associated with more likely to screen for anxiety | NR | NR | NR | NR | NR | No sig relationship between screening for anxiety cut-off and maternal or paternal occupation |
| Azim et al. 2019 | Yes | NR | NR | NR | NR | NR | NR | No sig relationship | NR | NR | NR | NR | NR | No sig relationship between anxiety scores and socioeconomic status ('good', 'moderate', or 'poor') | NR |
| Ballester et al. 2020* | No | NR | NR | NR | NR | NR | NR | NR | NR | NR | NR | NR | NR | NR | NR |
| Bantjes et al. 2019 * | No | NR | NR | NR | NR | NR | NR | NR | NR | NR | NR | NR | NR | NR | NR |
| Bassols et al. (2014) | Yes | NR | Females sig more likely to screen above the cut-off for anxiety | NR | NR | NR | NR | NR | Being in 1st yr sig associated with more likely to screen for anxiety | NR | NR | NR | No sig relationship between screening for anxiety cut-off and living arrangements | NR | No sig relationship between screening for anxiety cut-off and family income |
| Basudan et al. (2017) | Yes | Females sig higher scores | NR | NR | NR | NR | NR | No sig relationship | NR | NR | NR | NR | NR | NR | NR |
| Baykan et al. (2012) | No | NR | NR | NR | NR | NR | NR | NR | NR | NR | NR | NR | NR | NR | NR |
| Borst et al. (2015) | No | NR | NR | NR | NR | NR | NR | NR | NR | NR | NR | NR | NR | NR | NR |
| Bunevicius et al. (2008) | Yes | NR | NR | NR | NR | Humanities students had sig higher anxiety scores, compared to medical students | NR | NR | NR | NR | NR | NR | NR | NR | NR |
| Cheng et al. (2020) | Yes | NR | No sig gender diffs | NR | NR | NR | Medical students sig more likely to screen for anxiety, compared to Science & Technology and Literature & History students | NR | NR | NR | NR | NR | NR | NR | NR |
| Chernomas et al. (2013) | No | NR | NR | NR | NR | NR | NR | NR | NR | NR | NR | NR | NR | NR | NR |
| Cheung et al. (2016) | Yes | NR | No sig gender diffs | NR | Younger age group sig more likely to screen for anxiety | NR | NR | NR | No sig association between year of study and anxiety cut-offs | NR | NR | NR | NR | NR | NR |
| Cheung et al. (2020) | Yes | No sig gender diffs | NR | Sig positive relationship between age and anxiety score | NR | NR | General Nursing students sig more likely to screen for anxiety, compared to Mental Health Nursing students | NR | NR | NR | NR | NR | NR | NR | NR |
| Coker et al. (2018) | No | NR | NR | NR | NR | NR | NR | NR | NR | NR | NR | NR | NR | NR | NR |
| Dalky & Gharaibeh (2019) | Yes | Males sig higher scores | NR | NR | NR | NR | NR | NR | NR | NR | NR | NR | NR | NR | NR |
| Delara et al. (2015) | No | NR | NR | NR | NR | NR | NR | NR | NR | NR | NR | NR | NR | NR | NR |
| Eisenberg et al. (2007)^ | No | NR | NR | NR | NR | NR | NR | NR | NR | NR | NR | NR | NR | NR | NR |
| El-Gilany et al. (2019) | Yes | NR | NR | NR | Younger age group sig more likely to screen for anxiety | NR | NR | NR | Being in pre-clinical yrs sig associated with more likely to screen for anxiety | NR | NR | NR | Living outside campus sig more likely to screen above anxiety cut-off, compared to those living on campus or with family | NR | No sig relationship between screening for anxiety cut-off and socioeconomic standard ('very low/low', 'middle/high') |
| Eller et al. (2006) | Yes | NR | NR | Sig negative relationship between age and anxiety score | No association between age and anxiety cut-offs | NR | NR | Sig negative relationship between year of study and anxiety score | No sig association between year of study and anxiety cut-offs | NR | NR | NR | NR | NR | NR |
| El-Matury et al. (2018) | Yes | NR | No sig gender diffs | NR | No association between age and anxiety cut-offs | NR | No association between course of study and anxiety cut-offs | NR | NR | NR | NR | NR | NR | NR | NR |
| Fawzy & Hamed (2017) | Yes | Females sig higher scores | NR | Sig negative relationship between age and anxiety score | NR | NR | NR | Sig negative relationship: pre-clinical students had higher anxiety scores than clinical students | NR | NR | NR | Students who lived on campus had sig higher scores than students living with families | NR | NR | NR |
| Fernandes et al. (2018) | No | NR | NR | NR | NR | NR | NR | NR | NR | NR | NR | NR | NR | NR | NR |
| Fortney et al. 2016 | No | NR | NR | NR | NR | NR | NR | NR | NR | NR | NR | NR | NR | NR | NR |
| Francis et al. (2019) | Yes | No sig gender diffs | NR | NR | NR | NR | NR | Sig relationship found between years of study and anxiety scores, but not explained | NR | NR | NR | No sig relationship between anxiety scores and living arrangements | NR | No sig relationship between anxiety scores and family income ('low', 'middle', or 'high') | NR |
| Gaspersz et al. 2012 | No | NR | NR | NR | NR | NR | NR | NR | NR | NR | NR | NR | NR | NR | NR |
| Ge et al. (2020) | No | NR | NR | NR | NR | NR | NR | NR | NR | NR | NR | NR | NR | NR | NR |
| Islam et al. (2020) | Yes | NR | No sig gender diffs | NR | No association between age and anxiety cut-offs | NR | No association between course of study and anxiety cut-offs | NR | NR | NR | NR | NR | NR | NR | No sig relationship between screening for anxiety cut-off and paternal occupation or family income |
| Junaid et al. (2020) | Yes | NR | Females sig more likely to screen above the cut-off for anxiety | NR | NR | NR | NR | NR | Being in 4th yr sig associated with more likely to screen for anxiety | NR | NR | NR | NR | NR | NR |
| Karaoglu & Seker (2010) | Yes | No sig gender diffs | No sig gender diffs | NR | NR | NR | NR | No sig relationship | No sig association between year of study and anxiety cut-offs | NR | NR | NR | NR | Students sig more likely to have higher mean anxiety score if ≤ 1000YTL, compared to >1000YTL | Students sig more likely to screen for anxiety cut-off if ≤ 1000YTL, compared to >1000YTL |
| Kebede et al. (2020) | Yes | NR | Females sig more likely to screen above the cut-off for anxiety | NR | No association between age and anxiety cut-offs | NR | NR | NR | Being in 1st and 2nd yr sig associated with more likely to screen for anxiety | NR | NR | NR | NR | NR | NR |
| Knipe et al. (2018) | No | NR | NR | NR | NR | NR | NR | NR | NR | NR | NR | NR | NR | NR | NR |
| Kou et al. (2012) | No | NR | NR | NR | NR | NR | NR | NR | NR | NR | NR | NR | NR | NR | NR |
| Kulsoom & Afsar (2015) | Yes | No sig gender diffs | NR | NR | NR | NR | NR | Sig relationship found between years of study and anxiety scores, but not explained | NR | Saudi students sig reported higher mean anxiety scores compared to non-Saudi students | NR | No sig relationship between anxiety scores and living arrangements | NR | NR | NR |
| Kumar et al. (2019) | No | NR | NR | NR | NR | NR | NR | NR | NR | NR | NR | NR | NR | NR | NR |
| Kunwar et al. (2016) | Yes | NR | No sig gender diffs | NR | No association between age and anxiety cut-offs | NR | NR | NR | No sig association between year of study and anxiety cut-offs | NR | NR | NR | No sig relationship between screening for anxiety cut-off and living arrangements | NR | No sig relationship between screening for anxiety cut-off and socioeconomic standard ('low', 'middle', 'high') |
| Liu et al. (1997) | Yes | NR | No sig gender diffs | No sig relationship | NR | NR | NR | NR | No sig association between year of study and anxiety cut-offs | NR | NR | NR | NR | NR | NR |
| Lun et al. (2018) | Yes | NR | No sig gender diffs | NR | No association between age and anxiety cut-offs | NR | NR | NR | No sig association between year of study and anxiety cut-offs | NR | NR | NR | NR | NR | NR |
| Mahroon et al. (2018) | Yes | NR | Females sig more likely to screen above the cut-off for anxiety | NR | NR | NR | NR | NR | Being in 1st yr sig associated with more likely to screen for anxiety | NR | No sig difference between Arab and Non-Arab students | NR | No sig relationship between screening for anxiety cut-off and living arrangements | NR | NR |
| Marthoenis et al. (2018) | Yes | NR | Females sig more likely to screen above the cut-off for anxiety | NR | No association between age and anxiety cut-offs | NR | NR | NR | NR | NR | No sig difference between Achnese and Non-Achnese students | NR | No sig relationship between screening for anxiety cut-off and living arrangements | NR | NR |
| Milić et al. (2019) | Yes | NR | NR | No sig relationship | NR | NR | NR | NR | NR | NR | NR | NR | NR | NR | NR |
| Moutinho et al. (2017) | Yes | No sig gender diffs | NR | No sig relationship | NR | NR | NR | Sig negative relationship between year of study and anxiety score | NR | NR | NR | NR | NR | No sig relationship between anxiety scores and family income | NR |
| Moutinho et al. (2019) | No | NR | NR | NR | NR | NR | NR | NR | NR | NR | NR | NR | NR | NR | NR |
| Mundia (2010)^ | No | NR | NR | NR | NR | NR | NR | NR | NR | NR | NR | NR | NR | NR | NR |
| Nahm et al. (2020) | Yes | Females sig higher scores | NR | NR | NR | NR | NR | No sig relationship | NR | NR | NR | NR | NR | NR | NR |
| Nakhostin-Ansari et al. (2020) | Yes | NR | Females sig more likely to screen above the cut-off for anxiety | No sig relationship | NR | NR | NR | NR | No sig association between year of study and anxiety cut-offs | NR | NR | NR | No sig relationship between screening for anxiety cut-off and living arrangements | NR | NR |
| Naz et al. (2017) | Yes | NR | NR | NR | NR | NR | NR | NR | No sig association between year of study and anxiety cut-offs | NR | NR | NR | NR | NR | NR |
| Nimkuntod et al. (2016) | Yes | NR | NR | NR | NR | NR | NR | No sig relationship | NR | NR | NR | NR | NR | NR | NR |
| Paudel et al. (2020) | Yes | NR | Females sig more likely to screen above the cut-off for anxiety | NR | No association between age and anxiety cut-offs | NR | Sig difference between courses and anxiety cut-offs, but not explained | NR | NR | NR | NR | NR | NR | NR | Students whose fathers had<5 yrs education sig more likely to screen for anxiety-cut off than students whose fathers had >5 yrs education  No sig relationship between screening for anxiety cut-off and maternal education |
| Rab et al. (2008) | Yes | NR | NR | NR | NR | NR | NR | NR | No sig association between year of study and anxiety cut-offs | NR | NR | NR | No sig relationship between screening for anxiety cut-off and living arrangements | NR | NR |
| Ramón-Arbués et al. (2020) | Yes | Females sig higher scores | Females sig more likely to screen above the cut-off for anxiety | NR | Sig association between age and anxiety cut-offs: direction not described | NR | No association between course of study and anxiety cut-offs | NR | NR | NR | NR | NR | Sig difference found between students who lived alone or with friends, compared to family: directionality not described | NR | No sig relationship between screening for anxiety cut-off and perceived financial status ('low', 'medium', 'high') |
| Renteria et al. (2020)* | No | NR | NR | NR | NR | NR | NR | NR | NR | NR | NR | NR | NR | NR | NR |
| Saeed et al. (2017) | Yes | NR | Males sig more likely to screen above the cut-off for anxiety | NR | No association between age and anxiety cut-offs | NR | Pharmacy students sig more likely to screen for anxiety, compared to non-phramacy students | NR | NR | NR | NR | NR | NR | NR | NR |
| Sahoo & Khess (2010) | No | NR | NR | NR | NR | NR | NR | NR | NR | NR | NR | NR | NR | NR | NR |
| Salem et al. (2016) | Yes | NR | Females sig more likely to screen above the cut-off for anxiety | NR | Older age group sig more likely to screen for anxiety | NR | NR | NR | NR | NR | NR | NR | Students living with family sig more likely to screen above anxiety cut-off, compared to students in university housing or living with friends | NR | No sig relationship between screening for anxiety cut-off and perceived socioeconomic standard ('low', 'moderate', 'high') |
| Samaranayake et al. (2014) | Yes | NR | No sig gender diffs | NR | NR | NR | No association between course of study and anxiety cut-offs | NR | NR | NR | NR | NR | NR | NR | NR |
| Samson (2019) | No | NR | NR | NR | NR | NR | NR | NR | NR | NR | NR | NR | NR | NR | NR |
| Savitsky et al. (2020) | Yes | Females sig higher scores | No sig gender diffs | No sig relationship | NR | NR | NR | No sig relationship | NR | NR | NR | NR | NR | NR | NR |
| Serra et al. (2015) | Yes | NR | NR | NR | NR | NR | NR | NR | No sig association between year of study and anxiety cut-offs | NR | NR | NR | NR | NR | NR |
| Shawahna et al. (2020) | Yes | No sig gender diffs | NR | NR | NR | NR | NR | Sig negative relationship: pre-clinical students had higher anxiety scores than clinical students | NR | NR | NR | No sig relationship between anxiety scores and living arrangements | NR | NR | NR |
| Shen et al. (2020) | Yes | NR | No sig gender diffs | NR | Younger age group sig more likely to screen for anxiety | NR | NR | NR | NR | NR | Han students sig more likely to screen for anxiety compared to Non-Han students | NR | NR | NR | NR |
| Simić-Vukomanović et al. (2015) | Yes | NR | Females sig more likely to screen above the cut-off for anxiety | NR | No association between age and anxiety cut-offs | NR | Sig difference between courses and anxiety cut-offs, but not explained | NR | Being in 3rd yr sig associated with more likely to screen for anxiety | NR | NR | NR | NR | NR | Students from 'poor' and 'very poor' family economic situation sig more likely to screen for anxiety-cut off |
| Suarez et al. (2020) | No | NR | NR | NR | NR | NR | NR | NR | NR | NR | NR | NR | NR | NR | NR |
| Syed et al. (2018) | No | NR | NR | NR | NR | NR | NR | NR | NR | NR | NR | NR | NR | NR | NR |
| Tabalipa et al. (2015) | Yes | NR | Females sig more likely to screen above the cut-off for anxiety | NR | No association between age and anxiety cut-offs | NR | NR | NR | No sig association between being in first or second part of course and anxiety cut-offs | NR | NR | NR | NR | NR | No sig relationship between screening for anxiety cut-off and paternal education or maternal education |
| Tayefi et al. 2020 | Yes | NR | No sig gender diffs | NR | No association between age and anxiety cut-offs | NR | No association between course of study and anxiety cut-offs | NR | NR | NR | No sig difference between Persians, Turkic, Kurds, Lurs and Other | NR | No sig relationship between screening for anxiety cut-off and living arrangements | NR | No sig relationship between screening for anxiety cut-off and paternal education  Students whose mothers had tertiary-level education sig less likely to screen for anxiety-cut off than students whose mothers had dipolma or less level education |
| Teh et al. (2015) | Yes | No sig gender diffs | NR | NR | NR | NR | No association between course of study and anxiety cut-offs | NR | NR | Malay students sig more likely to screen for anxiety, compared to Chinese, Indian and Other | NR | NR | No sig relationship between screening for anxiety cut-off and living arrangements | NR | No sig relationship between screening for anxiety cut-off and monthly family income |
| Torres et al. 2017 | No | NR | NR | NR | NR | NR | NR | NR | NR | NR | NR | NR | NR | NR | NR |
| Umeh & Bangirana (2017) | Yes | No sig gender diffs | NR | Sig negative relationship between age and anxiety score | NR | NR | NR | NR | NR | NR | NR | NR | NR | NR | NR |
| Van Der Walt et al. (2020) | Yes | NR | Females sig more likely to screen above the cut-off for anxiety | NR | NR | NR | NR | NR | No sig association between year of study and anxiety cut-offs | NR | No sig differences between Black, White, Coloured, Indian, Asian or Other groups | NR | NR | NR | No sig relationship between screening for anxiety cut-off and monthly family income |
| Van Venrooij et al. (2017) | Yes | NR | No sig gender diffs | NR | NR | NR | NR | NR | No sig association between year of study and anxiety cut-offs | NR | NR | NR | No sig relationship between screening for anxiety cut-off and living arrangements | NR | NR |
| Verger et al. 2010 | No | NR | NR | NR | NR | NR | NR | NR | NR | NR | NR | NR | NR | NR | NR |
| Wang et al. (2020)^ | No | NR | NR | NR | NR | NR | NR | NR | NR | NR | NR | NR | NR | NR | NR |
| Wege et al. (2016) | Yes | NR | No sig gender diffs | NR | NR | NR | NR | NR | NR | NR | NR | NR | NR | NR | NR |
| Wong et al. (2006) | No | NR | NR | NR | NR | NR | NR | NR | NR | NR | NR | NR | NR | NR | NR |
| Wörfel et al. (2016) | Yes | NR | Females sig more likely to screen above the cut-off for anxiety | NR | NR | NR | No association between course of study and anxiety cut-offs | NR | Being in 5th yr sig associated with more likely to screen for anxiety | NR | NR | NR | NR | NR | NR |
| Zeng et al. (2019) | Yes | NR | No sig gender diffs | NR | No association between age and anxiety cut-offs | NR | NR | NR | NR | NR | NR | NR | NR | NR | No sig relationship between screening for anxiety cut-off and family economic situation ('good', 'poor') |

NR: Not reported in study.

* This study's data is also reported in Auerbach et al. (2016).

^Sample was mix of undergraduates and postgraduates: table reports data for undergraduates only.

**Appendix C**: Doi funnelplot for the 83 studies included in meta-analysis.


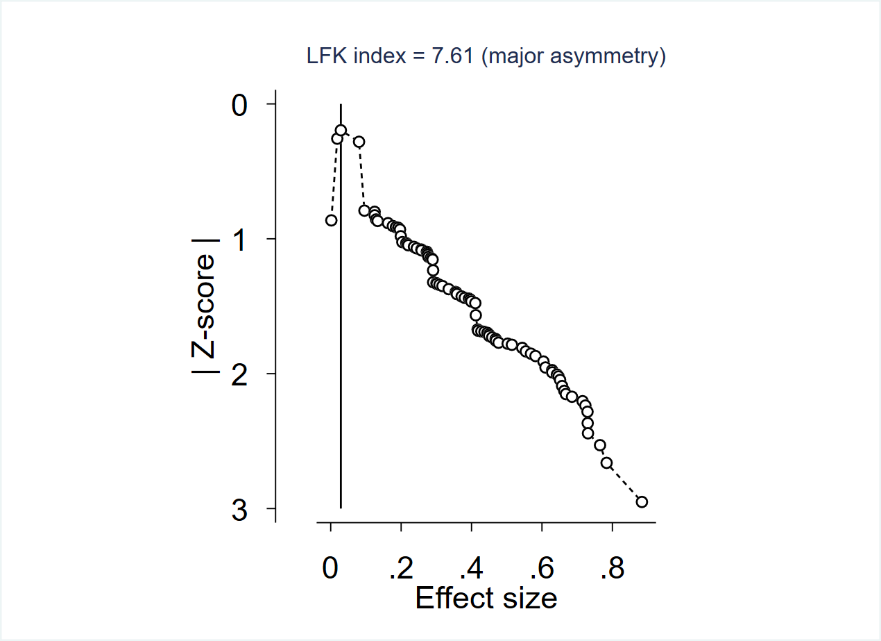


**Appendix D:** Full citations of the 89 studies included in the systematic review.

1. Abdallah, A., & Gabr, H. (2014). Depression, anxiety and stress among first year medical students in an Egyptian public university. International Research Journal of Medicine and Medical Sciences, 2(1), 11-19.

2. Abdel Wahed, W. Y., & Hassan, S. K. (2017). Prevalence and associated factors of stress, anxiety and depression among medical Fayoum University students. Alexandria Journal of Medicine, 53(1), 77-84. doi:https://doi.org/10.1016/j.ajme.2016.01.005

3. Abeetha, S., Sureka, Brinda, S., Ganesh, M., Olickal, J., & Sujatha, S. (2018). Prevalence of prehypertension and its association with levels of stress and anxiety among students of various disciplines in Chennai - A cross-sectional study. National Journal of Physiology, Pharmacy and Pharmacology, 8, 1599-1604.

4. Aboalshamat, K., Hou, X. Y., & Strodl, E. (2015). Psychological well-being status among medical and dental students in Makkah, Saudi Arabia: a cross-sectional study. Med Teach, 37 Suppl 1, S75-81. doi:10.3109/0142159x.2015.1006612

5. Al-Bahhawi T, Albasheer OB, Makeen AM, Arishi AM, Hakami OM, Maashi SM, et al. Depression, Anxiety, and Stress and Their Association with Khat Use: A Cross-Sectional Study among Jazan University Students, Saudi Arabia. Neuropsychiatr Dis Treat (2018) 14:2755-61. Epub 2018/11/15. doi: 10.2147/ndt.s182744

6. Al-Khani, A. M., Sarhandi, M. I., Zaghloul, M. S., Ewid, M., & Saquib, N. (2019). A cross-sectional survey on sleep quality, mental health, and academic performance among medical students in Saudi Arabia. BMC Res Notes, 12(1), 665. doi:10.1186/s13104-019-4713-2

7. Almhdawi, K. A., Kanaan, S. F., Khader, Y., Al-Hourani, Z., Almomani, F., & Nazzal, M. (2018). Study-related mental health symptoms and their correlates among allied health professions students. Work, 61(3), 391-401. doi:10.3233/wor-182815

8. AlShamlan, N. A., AlOmar, R. S., Al Shammari, M. A., AlShamlan, R. A., AlShamlan, A. A., & Sebiany, A. M. (2020). Anxiety and Its Association with Preparation for Future Specialty: A Cross-Sectional Study Among Medical Students, Saudi Arabia. J Multidiscip Healthc, 13, 581-591. doi:10.2147/jmdh.s259905

9. Alvi, T., Assad, F., Ramzan, M., & Khan, F. A. (2010). Depression, anxiety and their associated factors among medical students. J Coll Physicians Surg Pak, 20(2), 122-126.

10. Amir Hamzah, N. S., Nik Farid, N. D., Yahya, A., Chin, C., Su, T. T., Rampal, S. R. L., & Dahlui, M. (2019). The Prevalence and Associated Factors of Depression, Anxiety and Stress of First Year Undergraduate Students in a Public Higher Learning Institution in Malaysia. Journal of Child and Family Studies, 28(12), 3545-3557. doi:10.1007/s10826-019-01537-y

11. Asif, S., Mudassar, A., Shahzad, T. Z., Raouf, M., & Pervaiz, T. (2020). Frequency of depression, anxiety and stress among university students. Pak J Med Sci, 36(5), 971-976. doi:10.12669/pjms.36.5.1873

12. Auerbach, R. P., Alonso, J., Axinn, W. G., Cuijpers, P., Ebert, D. D., Green, J. G., . . . Bruffaerts, R. (2016). Mental disorders among college students in the World Health Organization World Mental Health Surveys. Psychol Med, 46(14), 2955-2970. doi:10.1017/s0033291716001665

13. Awadalla, S., Davies, E. B., & Glazebrook, C. (2020). A longitudinal cohort study to explore the relationship between depression, anxiety and academic performance among Emirati university students. BMC Psychiatry, 20(1), 448. doi:10.1186/s12888-020-02854-z

14. Azad, N., Shahid, A., Abbas, N., Shaheen, A., & Munir, N. (2017). Anxiety And Depression In Medical Students Of A Private Medical College. J Ayub Med Coll Abbottabad, 29(1), 123-127.

15. Azim, S. R., & Baig, M. (2019). Frequency and perceived causes of depression, anxiety and stress among medical students of a private medical institute in Karachi: a mixed method study. J Pak Med Assoc, 69(6), 840-845.

16. Ballester, L., Alayo, I., Vilagut, G., Almenara, J., Cebrià, A. I., Echeburúa, E., . . . Alonso, J. (2019). Accuracy of online survey assessment of mental disorders and suicidal thoughts and behaviors in Spanish university students. Results of the WHO World Mental Health- International College Student initiative. PLOS ONE, 14(9), e0221529. doi:10.1371/journal.pone.0221529

17. Bantjes, J., Lochner, C., Saal, W., Roos, J., Taljaard, L., Page, D., . . . Stein, D. J. (2019). Prevalence and sociodemographic correlates of common mental disorders among first-year university students in post-apartheid South Africa: implications for a public mental health approach to student wellness. BMC Public Health, 19(1), 922. doi:10.1186/s12889-019-7218-y

18. Bassols, A. M., Okabayashi, L. S., Silva, A. B. d., Carneiro, B. B., Feijó, F., Guimarães, G. C., . . . Eizirik, C. L. (2014). First- and last-year medical students: is there a difference in the prevalence and intensity of anxiety and depressive symptoms? Brazilian Journal of Psychiatry, 36, 233-240.

19. Basudan, S., Binanzan, N., & Alhassan, A. (2017). Depression, anxiety and stress in dental students. Int J Med Educ, 8, 179-186. doi:10.5116/ijme.5910.b961

20. Baykan Z, Naçar M, Cetinkaya F. Depression, Anxiety, and Stress among Last-Year Students at Erciyes University Medical School. Academic psychiatry : the journal of the American Association of Directors of Psychiatric Residency Training and the Association for Academic Psychiatry (2012) 36(1):64-5. Epub 2012/03/01. doi: 10.1176/appi.ap.11060125.

21. Borst JM, Frings-Dresen MH, Sluiter JK. Prevalence and Incidence of Mental Health Problems among Dutch Medical Students and the Study-Related and Personal Risk Factors: A Longitudinal Study. International journal of adolescent medicine and health (2016) 28(4):349-55. Epub 2016/11/05. doi: 10.1515/ijamh-2015-0021.

22. Bunevicius A, Katkute A, Bunevicius R. Symptoms of Anxiety and Depression in Medical Students and in Humanities Students: Relationship with Big-Five Personality Dimensions and Vulnerability to Stress. The International journal of social psychiatry (2008) 54(6):494-501. Epub 2008/11/01. doi: 10.1177/0020764008090843.

23. Chernomas WM, Shapiro C. Stress, Depression, and Anxiety among Undergraduate Nursing Students. International journal of nursing education scholarship (2013) 10. Epub 2013/11/10. doi: 10.1515/ijnes-2012-0032.

24. Cheng, S., Jia, C., & Wang, Y. (2020). Only Children Were Associated with Anxiety and Depressive Symptoms among College Students in China. Int J Environ Res Public Health, 17(11). doi:10.3390/ijerph17114035

25. Cheung, D. K., Tam, D. K. Y., Tsang, M. H., Zhang, D. L. W., & Lit, D. S. W. (2020). Depression, anxiety and stress in different subgroups of first-year university students from 4-year cohort data. Journal of Affective Disorders, 274, 305-314. doi:https://doi.org/10.1016/j.jad.2020.05.041

26. Cheung, T., Wong, S. Y., Wong, K. Y., Law, L. Y., Ng, K., Tong, M. T., . . . Yip, P. S. (2016). Depression, Anxiety and Symptoms of Stress among Baccalaureate Nursing Students in Hong Kong: A Cross-Sectional Study. Int J Environ Res Public Health, 13(8). doi:10.3390/ijerph13080779

27. Coker, A., Coker, O., & Sanni, D. (2018). Sociodemographic correlates and symptoms of depression, anxiety and stress among a sample of nigerian medical students. Nigerian Journal of Basic and Clinical Sciences, 15(1), 58-62. doi:10.4103/njbcs.njbcs_50_16

28. Dalky, H. F., & Gharaibeh, A. (2019). Depression, anxiety, and stress among college students in Jordan and their need for mental health services. Nurs Forum, 54(2), 205-212. doi:10.1111/nuf.12316

29. Delara M, Woodgate RL. Psychological Distress and Its Correlates among University Students: A Cross-Sectional Study. Journal of pediatric and adolescent gynecology (2015) 28(4):240-4. Epub 2015/05/31. doi: 10.1016/j.jpag.2014.08.012.

30. Eisenberg, D., Gollust, S. E., Golberstein, E., & Hefner, J. L. (2007). Prevalence and correlates of depression, anxiety, and suicidality among university students. Am J Orthopsychiatry, 77(4), 534-542. doi:10.1037/0002-9432.77.4.534

31. El-Gilany, A. H., Amro, M., Eladawi, N., & Khalil, M. (2019). Mental Health Status of Medical Students: A Single Faculty Study in Egypt. The Journal of nervous and mental disease, 207(5), 348-354. doi:10.1097/nmd.0000000000000970

32. El-Matury, H. J., Mardiah, B., Lestari, F., & Besral. (2018). Evaluation of Depression, Anxiety and Stress among Undergraduate Students in Jakarta. Indian Journal of Public Health Research & Development, 9(2), 296-301. doi:10.5958/0976-5506.2018.00135.3

33. Eller, T., Aluoja, A., Vasar, V., & Veldi, M. (2006). Symptoms of anxiety and depression in Estonian medical students with sleep problems. Depress Anxiety, 23(4), 250-256. doi:10.1002/da.20166

34. Fawzy, M., & Hamed, S. A. (2017). Prevalence of psychological stress, depression and anxiety among medical students in Egypt. Psychiatry Res, 255, 186-194. doi:10.1016/j.psychres.2017.05.027

35. Fernandes, M., Emanuelle, F., Vieira, R., Soares, J., Valéria, F., Avelino, F., & Santos, J. D. (2018). Prevalence of anxious and depressive symptoms in college students of a public institution. Revista brasileira de enfermagem, 71, 2169-2175. doi:10.1590/0034-7167-2017-0752

36. Fortney JC, Curran GM, Hunt JB, Cheney AM, Lu L, Valenstein M, et al. Prevalence of Probable Mental Disorders and Help-Seeking Behaviors among Veteran and Non-Veteran Community College Students. General hospital psychiatry (2016) 38:99-104. Epub 2015/11/26. doi: 10.1016/j.genhosppsych.2015.09.007.

37. Francis, B., Gill, J. S., Yit Han, N., Petrus, C. F., Azhar, F. L., Ahmad Sabki, Z., . . . Sulaiman, A. H. (2019). Religious Coping, Religiosity, Depression and Anxiety among Medical Students in a Multi-Religious Setting. Int J Environ Res Public Health, 16(2). doi:10.3390/ijerph16020259

38. Gaspersz R, Frings-Dresen MH, Sluiter JK. Prevalence of Common Mental Disorders among Dutch Medical Students and Related Use and Need of Mental Health Care: A Cross-Sectional Study. International journal of adolescent medicine and health (2012) 24(2):169-72. Epub 2012/08/23. doi: 10.1515/ijamh.2012.025.

39. Ge, F., Zhang, D., Wu, L., & Mu, H. (2020). Predicting Psychological State Among Chinese Undergraduate Students in the COVID-19 Epidemic: A Longitudinal Study Using a Machine Learning. Neuropsychiatric disease and treatment, 16, 2111-2118. doi:10.2147/NDT.S262004

40. Islam, S., Akter, R., Sikder, T., & Griffiths, M. D. (2020). Prevalence and Factors Associated with Depression and Anxiety Among First-Year University Students in Bangladesh: A Cross-Sectional Study. International Journal of Mental Health and Addiction. doi:10.1007/s11469-020-00242-y

41. Junaid, M., Ibn Auf, A., Shaikh, K., Khan, N., & Abdelrahim, S. (2020). Correlation between Academic Performance and Anxiety in Medical Students of Majmaah University - KSA. Journal of the Pakistan Medical Association, 70, 1. doi:10.5455/JPMA.19099

42. Karaoğlu, N., & Seker, M. (2010). Anxiety and Depression in Medical Students Related to Desire for and Expectations from a Medical Career. The West Indian medical journal, 59, 196-202.

43. Kebede, M. A., Anbessie, B., & Ayano, G. (2019). Prevalence and predictors of depression and anxiety among medical students in Addis Ababa, Ethiopia. International Journal of Mental Health Systems, 13(1), 30. doi:10.1186/s13033-019-0287-6

44. Knipe D, Maughan C, Gilbert J, Dymock D, Moran P, Gunnell D. Mental Health in Medical, Dentistry and Veterinary Students: Cross-Sectional Online Survey. BJPsych open (2018) 4(6):441-6. Epub 2018/11/20. doi: 10.1192/bjo.2018.61

45. Kou, C., Meng, X., Xie, B., Chen, Y., Yu, Q., Shi, J., . . . Huang, Y. (2012). The prevalence and correlates of neurotic disorders among undergraduates at a mainland Chinese university. Soc Psychiatry Psychiatr Epidemiol, 47(12), 2011-2018. doi:10.1007/s00127-012-0500-2

46. Kulsoom, B., & Afsar, N. A. (2015). Stress, anxiety, and depression among medical students in a multiethnic setting. Neuropsychiatr Dis Treat, 11, 1713-1722. doi:10.2147/ndt.s83577

47. Kumar, B., Shah, M. A. A., Kumari, R., Kumar, A., Kumar, J., & Tahir, A. (2019). Depression, Anxiety, and Stress Among Final-year Medical Students. Cureus, 11(3), e4257-e4257. doi:10.7759/cureus.4257

48. Kunwar, D., Risal, A., & Koirala, S. (2016). Study of Depression, Anxiety and Stress among the Medical Students in two Medical Colleges of Nepal. Kathmandu Univ Med J (KUMJ), 14(53), 22-26.

49. Liu, X. C., Oda, S., Peng, X., & Asai, K. (1997). Life events and anxiety in Chinese medical students. Social Psychiatry and Psychiatric Epidemiology, 32(2), 63-67. doi:10.1007/BF00788922

50. Lun, K. W., Chan, C. K., Ip, P. K., Ma, S. Y., Tsai, W. W., Wong, C. S., . . . Yan, D. (2018). Depression and anxiety among university students in Hong Kong. Hong Kong Med J, 24(5), 466-472. doi:10.12809/hkmj176915

51. Mahroon, Z. A., Borgan, S. M., Kamel, C., Maddison, W., Royston, M., & Donnellan, C. (2018). Factors Associated with Depression and Anxiety Symptoms Among Medical Students in Bahrain. Acad Psychiatry, 42(1), 31-40. doi:10.1007/s40596-017-0733-1

52. Marthoenis, Meutia, I., Fathiariani, L., & Sofyan, H. (2018). Prevalence of depression and anxiety among college students living in a disaster-prone region. Alexandria Journal of Medicine, 54(4), 337-340. doi:10.1016/j.ajme.2018.07.002

53. Milić, J., Škrlec, I., Milić Vranješ, I., Podgornjak, M., & Heffer, M. (2019). High levels of depression and anxiety among Croatian medical and nursing students and the correlation between subjective happiness and personality traits. Int Rev Psychiatry, 31(7-8), 653-660. doi:10.1080/09540261.2019.1594647

54. Moutinho ILD, Lucchetti ALG, Ezequiel ODS, Lucchetti G. Mental Health and Quality of Life of Brazilian Medical Students: Incidence, Prevalence, and Associated Factors within Two Years of Follow-Up. Psychiatry research (2019) 274:306-12. Epub 2019/03/05. doi: 10.1016/j.psychres.2019.02.041.

55. Moutinho, I. L. D., Maddalena, N. d. C. P., Roland, R. K., Lucchetti, A. L. G., Tibiriçá, S. H. C., Ezequiel, O. d. S., & Lucchetti, G. (2017). Depression, stress and anxiety in medical students: A cross-sectional comparison between students from different semesters. Revista da Associação Médica Brasileira, 63(1), 21-28.

56. Mundia, L. (2010). The Prevalence of Depression, Anxiety and Stress in Brunei Preservice Student Teachers. The Internet Journal of Mental Health, 6.

57. Nahm, S.-S., & Chun, M.-S. (2020). Stressors Predicting Depression, Anxiety, and Stress in Korean Veterinary Students. Journal of Veterinary Medical Education, e20190031. doi:10.3138/jvme-2019-0031

58. Nakhostin-Ansari, A., Sherafati, A., Aghajani, F., Khonji, M. S., Aghajani, R., & Shahmansouri, N. (2020). Depression and Anxiety among Iranian Medical Students during COVID-19 Pandemic. Iranian journal of psychiatry, 15(3), 228-235. doi:10.18502/ijps.v15i3.3815

59. Naz, N., Iqbal, S., & Mahmood, A. (2017). Stress, anxiety and depression among the Dental Students of University College of Medicine and Dentistry Lahore; Pakistan. Pakistan Journal of Medical and Health Sciences, 11, 1277-1281.

60. Nimkuntod, P., Uengarpon, N., Benjaoran, F., Pinwanna, K., Ratanakeereepun, K., & Tongdee, P. (2016). Psychometric Properties of Depression Anxiety and Stress in Preclinical Medical Students. J Med Assoc Thai, 99 Suppl 7, S111-117.

61. Paudel, S., Gautam, H., Adhikari, C., & Yadav, D. K. (2020). Depression, Anxiety and Stress among the Undergraduate Students of Pokhara Metropolitan, Nepal. Journal of Nepal Health Research Council, 18(1). doi:10.33314/jnhrc.v18i1.2189

62. Rab, F., Mamdou, R., & Nasir, S. (2008). Rates of depression and anxiety among female medical students in Pakistan. East Mediterr Health J, 14(1), 126-133.

63. Ramón-Arbués, E., Gea-Caballero, V., Granada-López, J. M., Juárez-Vela, R., Pellicer-García, B., & Antón-Solanas, I. (2020). The Prevalence of Depression, Anxiety and Stress and Their Associated Factors in College Students. Int J Environ Res Public Health, 17(19), 7001. doi:10.3390/ijerph17197001

64. Rentería, R., Benjet, C., Gutiérrez-García, R. A., Abrego-Ramírez, A., Albor, Y., Borges, G., . . . Mortier, P. (2020). Prevalence of 12-month mental and substance use disorders in sexual minority college students in Mexico. Soc Psychiatry Psychiatr Epidemiol. doi:10.1007/s00127-020-01943-4

65. Saeed, H., Saleem, Z., Razzaq, N., Akhtar, K., Maryam, A., Abbas, N., . . . Rasool, F. (2018). Determinants of Anxiety and Depression Among University Students of Lahore. International Journal of Mental Health and Addiction, 16. doi:10.1007/s11469-017-9859-3

66. Sahoo, S., & Khess, C. R. (2010). Prevalence of depression, anxiety, and stress among young male adults in India: a dimensional and categorical diagnoses-based study. The Journal of nervous and mental disease, 198(12), 901-904. doi:10.1097/NMD.0b013e3181fe75dc

67. Salem, G., Said, R., & Allah, M. (2016). Prevalence and Predictors of Depression, Anxiety and Stress among Zagazig University Students. The Medical journal of Cairo University, 84, 325-334.

68. Samaranayake, C. B., Arroll, B., & Fernando, A. T., 3rd. (2014). Sleep disorders, depression, anxiety and satisfaction with life among young adults: a survey of university students in Auckland, New Zealand. N Z Med J, 127(1399), 13-22.

69. Samson P. Role of Coping in Stress, Anxiety, Depression among Nursing Students of Purbanchal University in Kathmandu. Journal of Nepal Health Research Council (2019) 17(3):325-30. Epub 2019/11/19. doi: 10.33314/jnhrc.v17i3.1843

70. Savitsky, B., Findling, Y., Ereli, A., & Hendel, T. (2020). Anxiety and coping strategies among nursing students during the covid-19 pandemic. Nurse Educ Pract, 46, 102809. doi:10.1016/j.nepr.2020.102809

71. Serra, R., Dinato, S., & Caseiro, M. (2015). Prevalence of depressive and anxiety symptoms in medical students in the city of Santos. Jornal Brasileiro de Psiquiatria, 64, 213-220. doi:10.1590/0047-2085000000081

72. Shawahna, R., Hattab, S., Al-Shafei, R., & Tab’ouni, M. (2020). Prevalence and factors associated with depressive and anxiety symptoms among Palestinian medical students. BMC Psychiatry, 20(1), 244. doi:10.1186/s12888-020-02658-1

73. Shen, Y., Zhang, Y., Chan, B. S. M., Meng, F., Yang, T., Luo, X., & Huang, C. (2020). Association of ADHD symptoms, depression and suicidal behaviors with anxiety in Chinese medical college students. BMC Psychiatry, 20(1), 180. doi:10.1186/s12888-020-02555-7

74. Simic-Vukomanovic, I., Mihajlović, G., Kocic, S., Djonovic, N., Bankovic, D., Vukomanovic, V., & Djukic-Dejanovic, S. (2015). The prevalence and socioeconomic correlates of depressive and anxiety symptoms in a group of 1,940 Serbian university students. Vojnosanitetski pregled, 73, 143-143. doi:10.2298/VSP141106143S

75. Suarez DE, Cardozo AC, Ellmer D, Trujillo EM. Short Report: Cross Sectional Comparison of Anxiety and Depression Symptoms in Medical Students and the General Population in Colombia. Psychology, health & medicine (2021) 26(3):375-80. Epub 2020/04/22. doi: 10.1080/13548506.2020.1757130.

76. Syed, A., Ali, S. S., & Khan, M. (2018). Frequency of depression, anxiety and stress among the undergraduate physiotherapy students. Pakistan journal of medical sciences, 34(2), 468-471. doi:10.12669/pjms.342.12298

77. Tabalipa, F., Souza, M., Pfützenreuter, G., Lima, V., Traebert, E., & Traebert, J. (2015). Prevalence of Anxiety and Depression among Medical Students. Revista Brasileira de Educação Médica, 39(3), 388-394.

78. Tayefi, B., Eftekhar, M., Tayefi, M., Darroudi, S., Khalili, N., Mottaghi, A., . . . Nojomi, M. (2020). Prevalence and Socio-Demographic Correlates of Mental Health Problems Among Iranian Health Sciences Students. Acad Psychiatry, 44(1), 73-77. doi:10.1007/s40596-019-01121-y

79. Teh, C., Ngo, C., Zulkifli, R., Vellasamy, R., & Suresh, K. (2015). Depression, Anxiety and Stress among Undergraduate Students: A Cross Sectional Study. Open Journal of Epidemiology, 05, 260-268. doi:10.4236/ojepi.2015.54030

80. Torres, C., Otero, P., Bustamante, B., Blanco, V., Díaz, O., & Vázquez, F. L. (2017). Mental Health Problems and Related Factors in Ecuadorian College Students. Int J Environ Res Public Health, 14(5), 530. doi:10.3390/ijerph14050530

81. Umeh, G. C., & Bangirana, P. (2016). Cognitive and social predictors of generalized anxiety disorder symptoms among fresh undergraduates in Uganda. Tanzania Journal of Health Research, 19(1). doi:10.4314/thrb.v19i1.3

82. Van der Walt, S., Mabaso, W. S., Davids, E. L., & De Vries, P. J. (2019). The burden of depression and anxiety among medical students in South Africa: A cross-sectional survey at the University of Cape Town. S Afr Med J, 110(1), 69-76. doi:10.7196/SAMJ.2019.v110i1.14151

83. van Venrooij, L. T., Barnhoorn, P. C., Giltay, E. J., & van Noorden, M. S. (2015). Burnout, depression and anxiety in preclinical medical students: a cross-sectional survey. Int J Adolesc Med Health, 29(3). doi:10.1515/ijamh-2015-0077

84. Verger, P., Guagliardo, V., Gilbert, F., Rouillon, F., & Kovess-Masfety, V. (2010). Psychiatric disorders in students in six French universities: 12-month prevalence, comorbidity, impairment and help-seeking. Soc Psychiatry Psychiatr Epidemiol, 45(2), 189-199. doi:10.1007/s00127-009-0055-z

85. Wang, Z. H., Yang, H. L., Yang, Y. Q., Liu, D., Li, Z. H., Zhang, X. R., . . . Mao, C. (2020). Prevalence of anxiety and depression symptom, and the demands for psychological knowledge and interventions in college students during COVID-19 epidemic: A large cross-sectional study. J Affect Disord, 275, 188-193. doi:10.1016/j.jad.2020.06.034

86. Wege, N., Muth, T., Li, J., & Angerer, P. (2016). Mental health among currently enrolled medical students in Germany. Public Health, 132, 92-100. doi:10.1016/j.puhe.2015.12.014

87. Wong, J. G., Cheung, E. P., Chan, K. K., Ma, K. K., & Tang, S. W. (2006). Web-based survey of depression, anxiety and stress in first-year tertiary education students in Hong Kong. Aust N Z J Psychiatry, 40(9), 777-782. doi:10.1080/j.1440-1614.2006.01883.x

88. Wörfel, F., Gusy, B., Lohmann, K., Töpritz, K., & Kleiber, D. (2016). Mental health problems among university students and the impact of structural conditions. Journal of Public Health, 24(2), 125-133. doi:10.1007/s10389-015-0703-6

89. Zeng, Y., Wang, G., Xie, C., Hu, X., & Reinhardt, J. D. (2019). Prevalence and correlates of depression, anxiety and symptoms of stress in vocational college nursing students from Sichuan, China: a cross-sectional study. Psychol Health Med, 24(7), 798-811. doi:10.1080/13548506.2019.1574358
